# A Novel Candidate Vaccine for Cytauxzoonosis Inferred from Comparative Apicomplexan Genomics

**DOI:** 10.1371/journal.pone.0071233

**Published:** 2013-08-20

**Authors:** Jaime L. Tarigo, Elizabeth H. Scholl, David McK. Bird, Corrie C. Brown, Leah A. Cohn, Gregg A. Dean, Michael G. Levy, Denise L. Doolan, Angela Trieu, Shila K. Nordone, Philip L. Felgner, Adam Vigil, Adam J. Birkenheuer

**Affiliations:** 1 Center for Comparative Medicine and Translational Research, North Carolina State University, Raleigh, North Carolina, United States of America; 2 Bioinformatics Research Center, North Carolina State University, Raleigh, North Carolina, United States of America; 3 Department of Plant Pathology, North Carolina State University, Raleigh, North Carolina, United States of America; 4 Department of Pathology, University of Georgia, Athens, Georgia, United States of America; 5 Department of Veterinary Medicine and Surgery, University of Missouri, Columbia, Missouri, United States of America; 6 Department of Microbiology, Immunology, and Pathology, Colorado State University, Fort Collins, Colorado, United States of America; 7 Department of Population Health and Pathobiology, North Carolina State University, Raleigh, North Carolina, United States of America; 8 Division of Immunology, Queensland Institute of Medical Research, Queensland, Australia; 9 Department of Molecular Biomedical Sciences, North Carolina State University, Raleigh, North Carolina, United States of America; 10 Department of Medicine, University of California Irvine, Irvine, California, United States of America; 11 Department of Clinical Sciences, North Carolina State University, Raleigh, North Carolina, United States of America; Instituto Butantan, Brazil

## Abstract

Cytauxzoonosis is an emerging infectious disease of domestic cats (*Felis catus*) caused by the apicomplexan protozoan parasite *Cytauxzoon felis*. The growing epidemic, with its high morbidity and mortality points to the need for a protective vaccine against cytauxzoonosis. Unfortunately, the causative agent has yet to be cultured continuously *in vitro*, rendering traditional vaccine development approaches beyond reach. Here we report the use of comparative genomics to computationally and experimentally interpret the *C. felis* genome to identify a novel candidate vaccine antigen for cytauxzoonosis. As a starting point we sequenced, assembled, and annotated the *C. felis* genome and the proteins it encodes. Whole genome alignment revealed considerable conserved synteny with other apicomplexans. In particular, alignments with the bovine parasite *Theileria parva* revealed that a *C. felis* gene, cf76, is syntenic to p67 (the leading vaccine candidate for bovine theileriosis), despite a lack of significant sequence similarity. Recombinant subdomains of cf76 were challenged with survivor-cat antiserum and found to be highly seroreactive. Comparison of eleven geographically diverse samples from the south-central and southeastern USA demonstrated 91–100% amino acid sequence identity across cf76, including a high level of conservation in an immunogenic 226 amino acid (24 kDa) carboxyl terminal domain. Using *in situ* hybridization, transcription of cf76 was documented in the schizogenous stage of parasite replication, the life stage that is believed to be the most important for development of a protective immune response. Collectively, these data point to identification of the first potential vaccine candidate antigen for cytauxzoonosis. Further, our bioinformatic approach emphasizes the use of comparative genomics as an accelerated path to developing vaccines against experimentally intractable pathogens.

## Introduction

**Figure 1:**
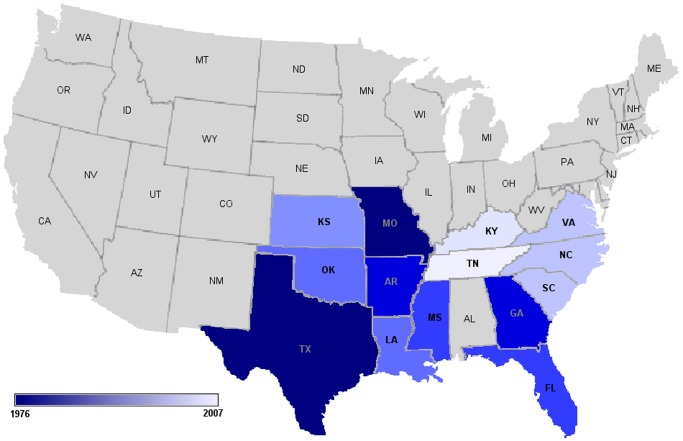
Distribution of cytauxzoonosis in the United States.

**Figure 2:**
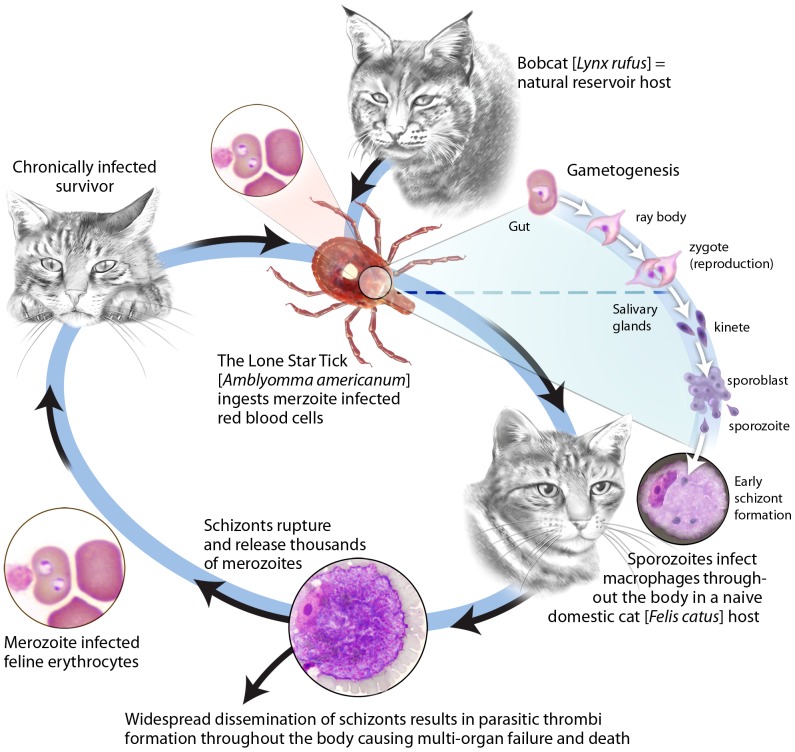
Life cycle of *Cytauxzoon felis*. The acute tissue stage of disease (the schizogenous phase) is characterized by wide spread dissemination of schizonts which form parasitic thrombi throughout the body resulting in a disease course that is typically fatal. Hosts that survive this acute tissue phase develop a chronic yet fairly innocuous erythroparasitemia with merozoite-infected red cells.

*Cytauxzoon felis* is a protozoan parasite of felids that causes cytauxzoonosis, an emerging disease in domestic cats. Without treatment nearly all cats die within three to five days of the onset of clinical symptoms. There are currently no effective means to prevent cytauxzoonosis, and even with treatment costing thousands of dollars, up to 40% of cats still succumb [1], [2]. First described in Missouri in 1976, the geographic range of *C. felis* is expanding and it has now been diagnosed in domestic cats in one third of US states (Figure 1) [1], [3], [4], [5], [6], [7], [8], [9], [10], [11]. Expansion of the geographic range is presumed to be due to changes in climate, urbanization, and increased exposure to the bobcat [*Lynx rufus*] reservoir host and the tick vector [*Amblyomma americanum*]. Bobcats experience a transient schizogenous tissue phase of limited pathogenicity followed by chronic erythroparasitemia. In domestic cats, the disease is usually characterized by a lethal acute schizogenous tissue phase. For animals that survive, a fairly innocuous chronic erythroparasitemia ensues (Figure 2). The high mortality, growing epidemic and cost of care point to vaccination as the most practical control strategy. Prior studies documenting the development of a protective immune response against *C. felis* imply that vaccine development is feasible [12], [13], [14], [15]. However the inability to culture *C. felis in vitro* has been a major barrier to discovery of protective antigens [14], and no vaccines against *C. felis* exist. In order to overcome experimental limitations and facilitate the rapid identification of vaccine candidate antigens we sequenced the entire 9.1 Mbp *C. felis* genome and identified approximately 4,300 protein-coding genes, each of which represents a potential protective antigen.

We used leading vaccine candidates from other apicomplexans as a guide to search for orthologues within the *C. felis* gene complement. *Cytauxzoon felis* is closely related to the apicomplexans *Theileria parva* and *Theileria annulata*, the etiologic agents of East Coast fever (ECF) and tropical theileriosis in cattle, respectively [16], [17], [18]. The leading vaccine candidate for *T. parva*, p67, has conferred substantial protection against ECF in clinical trials. Immunization of cattle with p67 reduced the incidence of severe ECF by 49% during field tick challenge trials in Kenya [19]. The *T. annulata* homologue of p67, SPAG-1, includes neutralizing epitopes on the carboxy terminus that are cross-reactive with p67, and SPAG-1 has been shown to confer protection to homologous species challenge [20], [21]. The functions of p67 and SPAG-1 have not been definitively identified although they are proposed to be involved in host cell recognition and invasion [20], [22].

Although *T. parva* p67 shares only 47% amino acid sequence identity with SPAG-1 [23], these two loci reside within a syntenic block of genes highly conserved between the two Theileria species, consistent with their orthology. We searched for *C. felis* orthologues of p67 and SPAG-1 but found no sequences with significant amino acid similarity. Therefore, guided by the approach used to identify the p67/SPAG-1 orthologue in *Babesia bovis* (BOV57), we used conserved genome synteny to expose the *C. felis* orthologue of p67/SPAG-1, which we call cf76 [24]. Here we report our assessment of three criteria likely to be important in determining suitability of cf76 as a vaccine candidate: 1) recognition by the feline immune system 2) degree of sequence similarity among *C. felis* isolates and 3) expression in the *C. felis* life stage that is believed to be critical for the development of a protective immune response.

## Materials and Methods

### Sequence and assembly of the *Cytauxzoon felis* genome and comparison of the *C. felis* genome with related apicomplexans

Whole blood (80 ml) was collected by sterile methods into citrate phosphate dextrose adenine (CPDA-1) anticoagulant immediately post-mortem from a domestic cat that died of acute *C. felis* infection. Acute infection was confirmed by microscopic observation of numerous *C. felis* schizonts in tissue imprints of liver, lung, and spleen. The blood was leuko-reduced to remove host nucleic acid contamination and isolate merozoite infected erythrocytes using a Purecell NEO Neonatal High Efficiency Leukocyte Reduction Filter for Red Cell Aliquots (PALL Corp., Port Washington, NY). *Cytauxzoon felis* genomic DNA was purified from leuko-reduced blood using the QIAamp DNA Blood Mini Kit (Qiagen, Valencia, CA).

We sequenced the *C. felis* genome using a 454 Genome Sequencer FLX (Roche, Indianapolis, IN) with Titanium chemistry and the standard Roche protocol. The sequence was assembled using Newbler 2.0 with a minimum overlap requirement of 90% identity over 30 bases. Resulting contigs were compared to the *Felis catus* genome and contaminating cat sequences (<2% of total reads) were removed.

*Cytauxzoon felis* tRNA and mRNA were isolated from purified merozoites. Whole blood (80 ml) was collected and leuko-reduced as described above. Following leuko-reduction, erythrocytes were lysed and tRNA and mRNA were purified using the Ribopure Blood Kit and PolyAPurist Mag Kit respectively (Ambion, Grand Island, NY) [25]. A cDNA library was constructed using the SMARTer PCR cDNA Synthesis Kit (Clontech, Mountain View, CA) and generation of expressed sequence tags (ESTs) was completed using a 454 Genome Sequencer FLX (Roche, Indianapolis, IN) with Titanium chemistry and the standard Roche protocol. ESTs were assembled with Newbler and the EST assembly was aligned to the genome using GeneDetective (Time Logic, Carlsbad, CA).

GeneMark-ES 2.5 (http://exon.gatech.edu/genemark_prok_gms_plus.cgi), which utilizes a Gibbs sampling algorithm to self-train for gene prediction, was deployed to create an initial computationally derived proteome. A combination of hand curated EST data and GeneMark results were used to create a training set for GlimmerHMM (http://www.cbcb.umd.edu/software/glimmer) to provide a second predicted proteome. Results from the EST comparisons, GlimmerHMM and GeneMark as well as sequence similarity searches against protein data from *B. bovis*, *T. parva*, *P. falciparum* and NCBI's non-redundant protein dataset were integrated into a generic Genome Browser (GBrowse). The *C. felis* genome and EST sequences are deposited with NCBI under BioProject PRJNA196611.

Predicted protein coding genes shared in common amongst the different organisms was determined. These were deduced by an all-against-all blastp search. Matches with at least 60% similarity across at least 30% of the query protein were accepted as matches. Proteins shared between multiple species were calculated based on the intersection of protein identifiers in pair-wise comparisons. No attempts were made to collapse paralogues.

### Identification and amplification *C. felis* cf76

**Figure 3:**
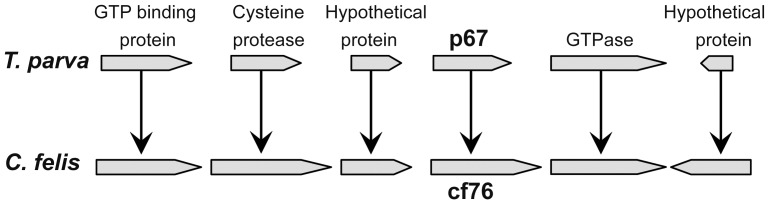
Conserved gene synteny between *T. parva* p67 and *C. felis* cf76. cf76 is identified *in silico* within a highly conserved syntenic block of genes similarly to the leading vaccine candidate for *T. parva*, p67.

A *C. felis* gene, cf76 (GenBank Accession KC986871), syntenic to p67/SPAG-1/BOV57 was identified *in silico*. p67, SPAG-1, and BOV57 are each located downstream of the same three highly conserved genes and upstream of the same two highly conserved genes (Figure 3). A BLAST search was used to identify the same highly conserved genes upstream and downstream of cf76. Amino acid and nucleotide alignments of cf76, p67, SPAG-1, and BOV57 were performed using clustalW [26]. Total RNA was extracted from *C. felis* merozoite and schizont-laden splenic tissue collected immediately post-mortem from a domestic cat that died of acute *C. felis* infection using the Trizol LS reagent (Sigma, St. Louis, MO), following the manufacturer's methods. Total RNA (10 µg/reaction) was treated twice with DNA-*free* DNase Treatment and Removal Reagent (Ambion, Grand Island, NY). Prior to generation of cDNA, the absence of contaminating DNA in the purified RNA was confirmed by PCR for *C. felis* 18S rRNA genes [3]. *Cytauxzoon felis* cDNA was produced using random hexamer primers (Promega, Madison WI) and Smartscribe reverse transcriptase (Clontech, Mountain View, CA). PCR to amplify the *C. felis* syntenic gene ORF with primers designed from the predicted flanking sequences was performed using previously published conditions with 25 pmol each of primer (5′ ATTGGATAGTAAATTAGGTTATAAG 3′ and 5′ GGAATTAATTCAGTTGGAATTTG 3′) and template (50 ng of *C. felis* splenic RNA, 1 µl of *C. felis* splenic cDNA, 16 ng of *C. felis* gDNA, or 1 µl of water) [3]. PCR products were analyzed by agarose gel electrophoresis. Identification of potential signal sequence and trans-membrane domains were deduced using Signal P Server v.4.0 and TMHMM Server v.2.0 from the Center for Biological Sequence Analysis. The GPI-anchor predictor PredGPI was employed to predict GPI-anchored protein sequence.

### Cloning and *in vitro* expression of cf76 and cf76 fragments

The cf76 ORF (2172 bp) and three overlapping subdomains of cf76 including the N-terminal region (720 bp), the central region (828 bp), and the C-terminal region (675 bp) were amplified from *C. felis* cDNA using primer pairs (Table 1) with a 20 bp adapter sequence at the 5′ and 3′ ends homologous to cloning sites of a linearized acceptor vector pXT7, to allow for directional cloning. PCR was performed with previously published conditions using 0.05 U/µl High Fidelity Expand Plus Taq DNA polymerase (Roche, Indianapolis, IN), 25 pmol of each primer (Table 1), and 5 ng of *C. felis* cDNA template [27], [28], [29].

**Table 1:** Primer sequences for amplification of cf76.

Primer	Sequence	Product
cf76 Forward ORF	5′ ACGACAAGCATATGCTCGAG- ATGAAATTTTTATTAATGTTTGTGGTGCCTTTG 3′	Full length cf76 (2172 bp)
cf76 Reverse ORF	5′ TCCGGAACATCGTATGGGTA- AACTAGTGTTAATGATAACAATAATGTAGC 3′	Full length cf76 (2172 bp)
cf76 Forward Fragment 1	5′ ACGACAAGCATATGCTCGAG-ATGAAATTTTTATTAATGTTTGTGGTGCCTTTG 3′	N-terminal region (720 bp)
cf76 Reverse Fragment 1	5′ TCCGGAACATCGTATGGGTA- TTCCACTTGAGGTCCAGTGACTATAC 3′	N-terminal region (720 bp)
cf76 Forward Fragment 2	5′ ACGACAAGCATATGCTCGAG-GATCGTGGCGGAAGTATAGTCACTG 3′	Central region (828 bp)
cf76 Reverse Fragment 2	5′ TCCGGAACATCGTATGGGTA- AGCTATTGAATGTTCTTCTTGTAATGAATT 3′	Central region (828 bp)
cf76 Forward Fragment 3	5′ ACGACAAGCATATGCTCGAG-GAAGAACATTCAATAGCTAATTCATTA 3′	C-terminal region (675 bp)
cf76 Reverse Fragment 3	5′ TCCGGAACATCGTATGGGTA- AACTAGTGTTAATGATAACAATAATGTAGC 3′	C-terminal region (675 bp)

Each amplified cf76 PCR product was cloned into a pXT7 vector containing an N-terminus 10× histidine (HIS) tag and a C-terminus hemagglutinin (HA) tag using homologous recombination as previously described [27], [28], [29] and all clones were sequenced bi-directionally. *In vitro* transcription and translation reactions (IVTT) were performed with purified recombinant plasmids using the RTS 100 E. coli HY kit (5 PRIME, Gaithersburg, MD).

### Purification of cf76 and cf76 subdomains

IVTT reaction components containing the entire cf76 protein and cf76 subdomains were purified using the N-terminal HIS tag under native and denaturing conditions with Qiagen Ni-NTA Magnetic Agarose Beads (Qiagen, Valencia, CA). Purity and quantity was assessed by western blot analysis in duplicate using secondary antibodies against the N-terminal HIS tag and the C-terminal HA tag using mouse anti-poly-HIS monoclonal IgG_2a_ antibody or mouse anti-poly-HA monoclonal antibody (Anti-His_6_ (2) and anti-HA clone 12CA5 respectively (Roche, Indianapolis, IN) as described below.

### SDS-PAGE and immunoblot analysis

cf76 and cf76 subdomain IVTT reactions and purified proteins were analyzed by western blot analysis. Proteins were subjected to SDS-PAGE (4–12% Bis-Tris Gel NuPAGE, Invitrogen, Grand Island, NY) and transferred onto polyvinylidene fluoride (PVDF) membranes (Millipore, Billerica, MA). After blocking (1× phosphate buffered saline containing 0.05% (v/v) Tween-20 (PBST), 2% nonfat milk, 2% bovine serum albumin (BSA), 2% gelatin from cold water fish skin for 1 h at room temperature, membranes were incubated with mouse anti-poly-HIS monoclonal antibody or mouse anti-poly-HA monoclonal antibody (Anti-His_6_ (2) and anti-HA clone 12CA5; Roche, Indianapolis, IN) overnight at 4°C. After three consecutive washes for 5 min at room temperature in PBST, membranes were incubated with horse radish peroxidase (HRP) conjugated goat anti-mouse immunoglobulin (H+L IgG; Biorad, Hercules, CA) at room temperature for 1 h and washed 3× with PBST at room temperature. Immobilon Western Chemiluminescent HRP Substrate (Millipore, Billerica, MA) was used for signal detection.

The immune response to purified cf76 and cf76 subdomains was assessed by western blotting using pooled sera from 10 domestic cats that survived natural *C. felis* infection, as well as 10 näive cats. To determine *C. felis* infection status, genomic DNA (gDNA) was purified using the QIAamp DNA Blood Mini Kit (Qiagen, Valencia, CA) and real-time PCR for *C. felis* 18S and for the feline house-keeping gene GAPDH was performed using previously published methods [3], [30]. Western blots of purified protein were prepared and blocked as described with the addition of 5% goat serum. Cat sera was diluted 1∶500 in blocking buffer containing 1.5 mg/ml *E. coli* lysate (MCLAB, South San Francisco, CA) and incubated for 2 h at room temperature to adsorb *E. coli* binding proteins in feline sera that may contribute to background. Membranes were incubated with pre-adsorbed cat sera for 2 h at room temperature, washed 5×5 min in PBST, incubated for 1 h at room temperature with goat anti-cat HRP antibody (H+L IgG; Jackson ImmunoResearch, West Grove, PA), washed 5×5 min in PBST, and a chemiluminescent signal was detected with a luminometer (Perkin Elmer, Massachusetts, NY).

### Conservation of cf76 sequence from diverse geographic regions

Genomic DNA was extracted from 11 *C. felis* infected whole blood samples collected from geographically diverse regions using QIAamp DNA Blood Mini Kit (Qiagen, Valencia, CA). cf76 was amplified by PCR under the following conditions: 0.05 U/µl High Fidelity Expand Plus Taq DNA polymerase (Roche, Indianapolis, IN), 0.2 mM of each dNTP, 1× reaction buffer, 25 pmol of each primer (FOR- 5′ ATTGGATAGTAAATTAGGTTATAAG 3′ and REV- 5′ GGAATTAATTCAGTTGGAATTTG 3′), 5 µl gDNA, initial denaturation at 95°C for 5 min; 40 cycles of 95°C for 30 sec, 54°C for 1.5 min, and 72°C for 2 min; and a final extension at 72°C for 10 min. The cf76 sequences from these samples (GenBank Accessions KC986861–KC986871) and sequences from *B. bovis* (BOV57, GenBank FJ805276.1, ACY08791.1), *T. parva* (p67, GenBank U40703.1, AAB06703.1) and *T. annulata* (SPAG-1, GenBank M63017.1, AAA30134.1) were aligned (http://www.ebi.ac.uk/Tools/msa/clustalw2/).

For tree construction, DNA sequences for the 11 cf76 sequences were aligned using ClustalX [26]. Phylogenetic reconstruction was performed via MrBayes [31] using a GTR model with gamma-distributed rate variation across sites. A total of 100,000 generations were run on 2 chains with a sample frequency of 100. A burn-in of 100 data points was more than sufficient to reach convergence.

### Transcription of cf76 in schizonts

*C. felis* infected lung tissues were harvested and formalin fixed immediately post-mortem from a cat that died of acute cytauxzoonosis. Hematoxylin and eosin (H&E) stained sections were examined for the presence of schizonts. The C-terminal region (678 bp) of cf76 was amplified by PCR, cloned into the pGEM-T Easy vector (Promega, Madison, WI) and sequenced bi-directionally. A negative sense digoxigenin-labeled riboprobe was generated and *in situ* hybridization was performed as previously described on infected lung tissue including the use of an non-specific (avian viral pathogen derived sequence) digoxigenin-labeled negative sense probe [32].

### Ethics Statement

This study was conducted in strict accordance with the recommendations of North Carolina State University Institutional Animal Care and Use Committee (NCSU IACUC) approved protocol 09-067-O. The samples used for this publication were either scheduled to be discarded from of a previous study (collected with owner consent under NCSU IACUC approved protocol 09-067-O) or were diagnostic samples that were scheduled to be discarded.

## Results and Discussion

### Sequence and assembly of the *Cytauxzoon felis* genome and comparison of the *C. felis* genome with related apicomplexans

The *C. felis* sequence assembled into 361 contigs spanning 9.1 mega-bases (MB) of genomic DNA post decontamination of *F. catus* sequence (Table 2). The largest contiguous stretch of genomic sequence was 183 kb, with an N_50_ of greater than 70 kb. This genomic data was used to establish an initial computationally predicted proteome of 4,323 genes using the self-training program GeneMark.hmm-ES (v2.5).

**Table 2:** Sequence assembly of *Cytauxzoon felis* genomic and cDNA.

	Genomic	ESTs^a^
**Raw Reads**	603,160	202,774
**Feline Contamination (%)**	4.9	53.9
***C. felis*** **Contigs**	361	962
**Total # Bases in Contigs**	9,110,259	547,540
**Largest Contig (bp)**	183,236	4,132
**Contig N50** b	70,451	639
**G+C Composition (%)**	31.8	36.2

a Expressed Sequence Tags.

b The contig length such that using equal or longer contigs produces half the bases of the assembly.

The *C. felis* EST data assembled to 962 contigs covering 547 kb of gene space (Table 2). These contigs were used in a BLASTX search against the NCBI non-redundant database to identify contigs that likely represent close to full-length genes. A GeneDetective search of the ESTs against the genomic data provided information about gene structure (Time Logic, Carlsbad, CA).

A set of 100 randomly selected GeneMark predictions and 57 hand-curated full-length ESTs were then used as a training set for GlimmerHMM (v.3.02). Based on that training set, GlimmerHMM predicted 4,378 genes (Table 3). Although there was some slight variation between the two computationally derived gene sets (Table 3), approximately 25% of the genes are identical between the two, and a further 50% differ only in ascribing the most 5′ or most 3′ exons; such discrepancies were typically straightforward to resolve with manual curation.

**Table 3:** Comparison of gene predictions of the *Cytauxzoon felis* genome with related apicomplexans.

	*C. felis*		*T. parva* ^23^	*B. bovis* ^23^	*P. falciparum* ^23^
	GeneMark	Glimmer			
**Genome Size (Mbp)**	9.1	9.1	8.3	8.2	22.8
**G+C Composition (%)**	31.8	31.8	34.1	41.8	19.4
**Protein Coding Genes**	4,323	4,378	4,061	3,706	5,337
**Average Protein (aa)**	466	409	469	505	761
**% Genes with Introns**	68.7	61.7	73.6	61.5	53.9
**Protein Coding (%)**	66.2	68.8	68.4	70.2	52.6

In comparing the *C. felis* genome (GenBank Accession PRJNA196611) to the genomes of three related apicomplexans, *T. parva* (GenBank Accession PRJNA16136), *B. bovis* (GenBank Accession PRJNA20343) and *P. falciparum* (GenBank Accession PRJNA148) attributes such as genome size, %GC content, average protein length and number of protein-coding genes most closely resemble *T. parva* and are most different from *P. falciparum* (Table 3). A comparison of predicted genes between these sets reveals more similar genes in common with *T. parva*. A total of 914 similar genes are present in all four apicomplexans, and 2,420 are shared by *C. felis*, *B. bovis* and *T. parva* but are not found in *P. falciparum*. Note that the numbers in each sector of the Venn Diagram are not strictly additive due to the variation in size of different gene families within each of the respective genomes, but provide an overall indication as to the relatedness between the organisms as a whole (Figure S1).

### Identification and characterization of *C. felis* cf76

Given that *C. felis* is most closely related to *Theileria* spp, a BLAST search was used to identify a *C. felis* orthologue to p67/SPAG-1. However, no *C. felis* genes with significant identity to p67 or SPAG-1 were identified within the *C. felis* genome. Therefore we used genome synteny as a guide, and identified a 2172 bp single copy *C. felis* gene syntenic to p67. *Theileria parva* p67, *T. annulata* SPAG-1, and *B. bovis* BOV57 antigens are encoded by genes that reside within a syntenic block that is highly conserved between the three species and a similar syntenic block of *C. felis* genes was identified *in silico* (Figure 3). cf76, p67, SPAG-1, and BOV57 are 723aa, 752aa, 907aa, and 494aa in length respectively. The *C. felis* isolate submitted for genome sequencing was slightly larger than that of the majority of geographically diverse isolates sequenced which were approximately 706aa. Similar to BOV57, cf76 contains a single exon, while p67 and SPAG-1 contain two exons. Consistent with the BLAST result, cf76 only shared 22%, 23%, and 23% nucleotide identities with p67, SPAG-1, and BOV57 respectively (Figure S2) and 13%, 13%, and 14% amino acid similarities with p67, SPAG-1, and BOV57 (Figure S3). Based on the mean predicted molecular weight across isolates sequenced (75,557.78 Da) we designated the gene that is syntenic to p67/SPAG-1/BOV57 as cf76.

Similar to p67, SPAG-1, and BOV57, cf76 encodes a protein with a predicted signal peptide sequence at the amino terminus, suggesting that this protein may be secreted. In contrast to p67/SPAG-1, cf76 does not have a transmembrane domain, suggesting that it is unlikely to be membrane bound. Also unique to cf76 is a putative glycosylphosphatidylinositol (GPI) anchor. GPI anchors are glycolipids that anchor membrane proteins and have been associated with immunoreactivity in some protozoan pathogens [33]. While it is difficult to definitively establish orthology of cf76 with p67/SPAG-1 using sequence identity, we speculate that conserved synteny combined with a lack of conserved sequence identity may indicate an orthologous gene that is under extreme pressure from the host immune response.

### Feline humoral immune response to recombinant cf76

**Figure 4:**
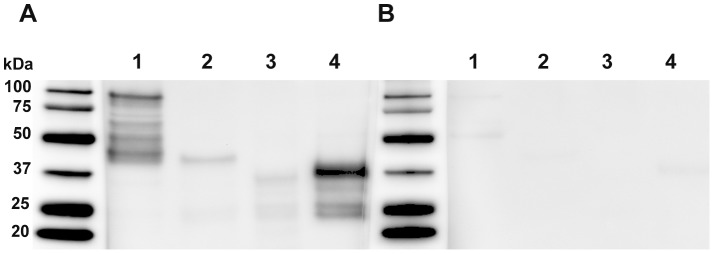
Assessment of feline sero-reactivity to cf76 and cf76 fragments by western blot. Purified full length cf76 (1), the N-terminal region (2), the central region (3), and the C-terminal region (4) were probed with pooled sera (1∶500) from cats surviving *C. felis* infection (A) or naive cats (B).

**Figure 5:**
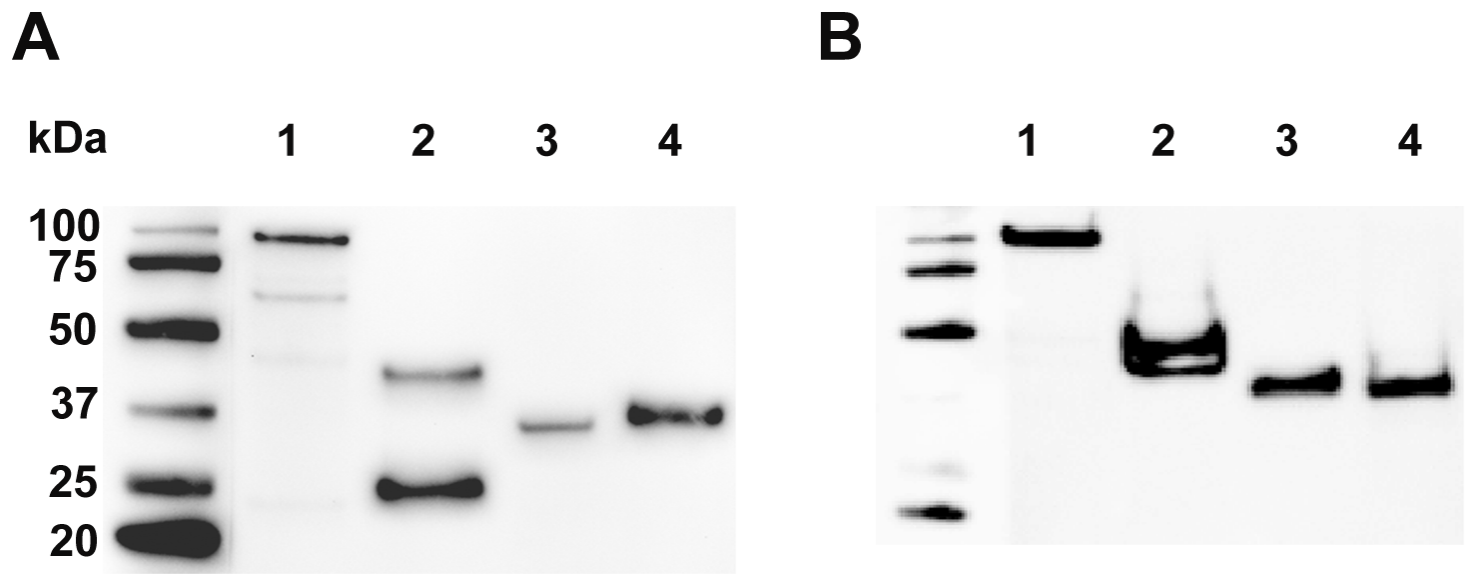
Assessment of purified cf76 and cf76 fragments by western blot. Purified full length cf76 (1), the N-terminal region (2), the central region (3), and the C-terminal region (4) were probed with anti-HIS N-terminal tag (A) and anti-HA C-terminal tag antibodies (B).

Western blot analysis using pooled sera from 10 cats surviving *C. felis* infection revealed strong seroreactivity to HIS-purified recombinant cf76 and the C-terminal region. In comparison, lower intensity signal was detected against the central and N-terminal regions of cf76 with immune sera. Substantial reactivity was not observed using pooled sera from 10 cats that tested negative for *C. felis*, and observed signal was attributed to low levels of cross-reacting antibodies unrelated to *C. felis* infection (Figure 4). The apparent molecular mass of full length cf76, the N-terminal region, the central region, and the C-terminal region were approximately 100 kDa, 42 kDa, 35 kDa and 37 kDa, despite predicted molecular mass of 81.6 kDa, 27.1 kDa, 33 kDa, and 26.8 kDa respectively (Figure 5). Production and co-purification of partial transcripts as well as putative degradation products were observed for western blots probed with anti-HIS antibodies while only complete proteins were observed on blots probed with anti-HA antibodies. Collectively these data support that the C-terminus of cf76 is highly immunogenic during natural infection with *C. felis*. Future experiments evaluating sera from individual cats will provide more information on the percentage of cats surviving *C. felis* infection that have robust antibody responses against cf76.

### cf76 sequence is conserved between samples from different geographic regions

In order to assess the degree of conservation amongst *C. felis* parasite samples from different geographic regions, we amplified and sequenced cf76 from eleven different samples from eight states in the southeastern and south-central United States, revealing a high degree of conservation (92.2 to 100% identity) (Figure S4). Evidence of phylogenetic divergence is seen between the isolates obtained from the two states furthest east (NC and VA) and the other states (Figure S5). Preliminary epitope mapping revealed that high levels of feline antibodies are developed against linear epitopes present in the C-terminal region (Figure 4). This region is highly conserved amongst samples. The only variation in this region was that ten of eleven samples had a tandem repeat of 30 bp sequence while the remaining sample only had this 30 bp sequence once.

### cf76 is expressed in the *C. felis* life-stage associated with immune protection

**Figure 6:**
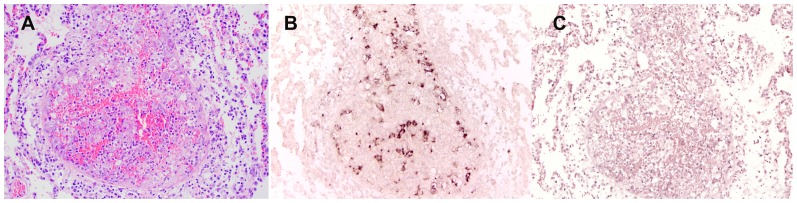
In situ hybridization to identify transcription of cf76 in C. felis-infected lung tissue. A. Hematoxylin and eosin stained lung tissue demonstrating schizonts forming a parasitic thrombus within a pulmonary vessel, 20X, B. Negative sense riboprobe, hematoxylin and eosin counterstain, numerous positive cells (brown) are demonstrating intracytoplasmic presence of cf76 antigen, 20X, C. Irrelevant negative sense riboprobe, hematoxylin and eosin counterstain, 20X.

*Cytauxzoon* spp. has a complex life cycle with three life stages in the mammalian host: sporozoites, schizonts, and merozoites (Figure 2). Of these, schizonts have been associated with a protective immune response. Solid immunity to *C. felis* was observed in cats that had previously survived the schizogenous phase of cytauxzoonosis [12], [14], [34]. These cats survived challenge infection with no signs of illness while naïve control cats died of cytauxzoonosis. In contrast direct inoculation with *C. felis* merozoites alone has not conferred protective immunity. Collectively, these data suggest antigens associated with schizonts are vaccine targets for *C. felis*. Based on these findings we investigated expression of cf76 in the schizont stage of *C. felis* using *in situ* hybridization. Using a labeled negative sense riboprobe directed against cytoplasmic mRNA, we found robust levels of cf76 transcripts in the schizogenous tissue stage of *C. felis* (Figure 6) further supporting consideration of this antigen as a vaccine candidate.

## Conclusions

Prior to our work no protein coding genes from *C. felis* had been characterized. Based on a full genome sequence we have now identified ∼4,300 protein coding genes and characterized the first vaccine candidate for *C. felis*. Specifically, our work demonstrates the potential of cf76 as a vaccine candidate antigen for cytauxzoonosis as it is: 1) recognized by the feline humoral immune system, 2) highly conserved amongst isolates and 3) transcribed in the life stage of *C. felis* shown to confer protective immunity. To substantiate the efficacy of cf76 as a vaccine antigen, significant reduction in morbidity and mortality of cytauxzoonosis must be demonstrated in immunization and challenge trials.

Our bioinformatic approach provides an example of how comparative genomics can provide an accelerated path to identify vaccine candidates in experimentally intractable pathogens. In addition to identification of specific candidate genes, this approach provides a valuable resource for future comparative genomic and proteomic studies to accelerate identification of additional vaccine candidates and drug targets for *C. felis* and related apicomplexans.

## Supporting Information

Figure S1 **Four way Venn Diagram: Protein coding genes of** ***Cytauxzoon felis*** **and related apicomplexan parasites.**
(TIF)

Figure S2 **Nucleotide sequence alignment of syntenic genes** ***C. felis*** **cf76-1 (isolate from the** ***C. felis*** **genome sequence), cf76-2 (the most common sequence among eleven geographically diverse** ***C. felis*** **isolates),** ***T. parva*** **p67 (GenBank U40703.1),** ***T. annulata*** **SPAG-1 (GenBank M63017.1), and** ***B. bovis*** **BOV57 (GenBank FJ805276.1).**
(DOC)

Figure S3 **Amino acid sequence alignment of syntenic genes** ***C. felis*** **cf76-1 (isolate from the** ***C. felis*** **genome sequence), cf76-2 (the most common sequence among eleven geographically diverse** ***C. felis*** **isolates),** ***T. parva*** **p67 (GenBank AAB06703.1),** ***T. annulata*** **SPAG-1 (GenBank AAA30134.1), and** ***B. bovis*** **BOV57 (GenBank ACY08791.1) (shading: black- identical amino acids, grey- similar amino acids).**
(DOC)

Figure S4 **Amino acid sequences of syntenic gene** ***C. felis*** **cf76 from geographic isolates across the southeastern and southwestern United States (shading: black- identical amino acids, grey- similar amino acids).**
(DOC)

Figure S5 **Phylogenetic reconstruction of cf76 across 11 isolates.** Posterior probability of clades is indicated on branches. Sample names are based on the name of the individual animal and the state from which the sample was taken (AR - Arkansas, KS - Kansas, MO - Missouri, NC - North Carolina, OK - Oklahoma, OH - Ohio, TN - Tennessee, VA - Virginia). Scale bar indicates expected changes per site. Full genome sequence is derived from Winnie-VA.
(TIF)

## References

[1] Birkenheuer AJ, Le JA, Valenzisi AM, Tucker MD, Levy MG, et al (2006) *Cytauxzoon felis* infection in cats in the mid-Atlantic states: 34 cases (1998–2004). J Am Vet Med Assoc 228: 568–571. 16478435 10.2460/javma.228.4.568

[2] Cohn LA, Birkenheuer AJ, Brunker JD, Ratcliff ER, Craig AW (2011) Efficacy of atovaquone and azithromycin or imidocarb dipropionate in cats with acute cytauxzoonosis. J Vet Intern Med 25: 55–60. 21143646 10.1111/j.1939-1676.2010.0646.x

[3] Birkenheuer AJ, Marr H, Alleman AR, Levy MG, Breitschwerdt EB (2006) Development and evaluation of a PCR assay for the detection of *Cytauxzoon felis* DNA in feline blood samples. Vet Parasitol 137: 144–149. 16417970 10.1016/j.vetpar.2005.12.007

[4] Birkenheuer AJ, Marr HS, Warren C, Acton AE, Mucker EM, et al (2008) *Cytauxzoon felis* infections are present in bobcats (Lynx rufus) in a region where cytauxzoonosis is not recognized in domestic cats. Vet Parasitol 153: 126–130. 18295403 10.1016/j.vetpar.2008.01.020

[5] Glenn BL, Stair EL (1984) Cytauxzoonosis in domestic cats: report of two cases in Oklahoma, with a review and discussion of the disease. J Am Vet Med Assoc 184: 822–825. 6725117

[6] Haber MD, Tucker MD, Marr HS, Levy JK, Burgess J, et al (2007) The detection of *Cytauxzoon felis* in apparently healthy free-roaming cats in the USA. Vet Parasitol 146: 316–320. 17391852 10.1016/j.vetpar.2007.02.029

[7] Hauck WN, Snider TG (1982) Cytauxzoonosis in a native Louisiana cat. J Am Vet Med Assoc 180: 1472–1474. 7201462

[8] Jackson CB, Fisher T (2006) Fatal cytauxzoonosis in a Kentucky cat (*Felis domesticus*). Vet Parasitol 139: 192–195. 16584845 10.1016/j.vetpar.2006.02.039

[9] Kier A, Morehouse L, Wagner J (1979) Feline cytauxzoonosis: An update. Missouri Vet 29: 15–18.

[10] Meier HT, Moore LE (2000) Feline cytauxzoonosis: a case report and literature review. J Am Anim Hosp Assoc 36: 493–496. 11105885 10.5326/15473317-36-6-493

[11] Wagner JE (1976) A fatal cytauxzoonosis-like disease in cats. J Am Vet Med Assoc 168: 585–588. 818065

[12] Ferris DH (1979) A progress report on the status of a new disease of American cats: cytauxzoonosis. Comp Immunol Microbiol Infect Dis 1: 269–276. 122558 10.1016/0147-9571(79)90028-6

[13] Motzel SL, Wagner JE (1990) Treatment of experimentally induced cytauxzoonosis in cats with parvaquone and buparvaquone. Vet Parasitol 35: 131–138. 2111598 10.1016/0304-4017(90)90122-r

[14] Shindel N, Dardiri AH, Ferris DH (1978) An indirect fluorescent antibody test for the detection of Cytauxzoon-like organisms in experimentally infected cats. Can J Comp Med 42: 460–465. 369664 PMC1277671

[15] Uilenberg G, Franssen FF, Perie NM (1987) Relationships between *Cytauxzoon felis* and African piroplasmids. Vet Parasitol 26: 21–28. 3125663 10.1016/0304-4017(87)90073-2

[16] Ketz-Riley CJ, Reichard MV, Van den Bussche RA, Hoover JP, Meinkoth J, et al (2003) An intraerythrocytic small piroplasm in wild-caught Pallas's cats (*Otocolobus manul*) from Mongolia. J Wildl Dis 39: 424–430. 12910772 10.7589/0090-3558-39.2.424

[17] Criado-Fornelio A, Gonzalez-del-Rio MA, Buling-Sarana A, Barba-Carretero JC (2004) The “expanding universe” of piroplasms. Vet Parasitol 119: 337–345. 15154598 10.1016/j.vetpar.2003.11.015

[18] Lack JB, Reichard MV, Van Den Bussche RA (2012) Phylogeny and evolution of the Piroplasmida as inferred from 18S rRNA sequences. Int J Parasitol 42: 353–363. 22429769 10.1016/j.ijpara.2012.02.005

[19] Musoke A, Rowlands J, Nene V, Nyanjui J, Katende J, et al (2005) Subunit vaccine based on the p67 major surface protein of *Theileria parva* sporozoites reduces severity of infection derived from field tick challenge. Vaccine 23: 3084–3095. 15811656 10.1016/j.vaccine.2004.09.039

[20] Boulter N, Knight PA, Hunt PD, Hennessey ES, Katzer F, et al (1994) *Theileria annulata* sporozoite surface antigen (SPAG-1) contains neutralizing determinants in the C terminus. Parasite Immunol 16: 97–104. 7517029 10.1111/j.1365-3024.1994.tb00328.x

[21] Hall R, Boulter NR, Brown CG, Wilkie G, Kirvar E, et al (2000) Reciprocal cross-protection induced by sporozoite antigens SPAG-1 from *Theileria annulata* and p67 from *Theileria parva* . Parasite Immunol 22: 223–230. 10792761 10.1046/j.1365-3024.2000.00302.x

[22] Shaw MK, Tilney LG, Musoke AJ (1991) The entry of *Theileria parva* sporozoites into bovine lymphocytes: evidence for MHC class I involvement. J Cell Biol 113: 87–101. 1901066 10.1083/jcb.113.1.87 PMC2288915

[23] Brayton KA, Lau AO, Herndon DR, Hannick L, Kappmeyer LS, et al (2007) Genome sequence of *Babesia bovis* and comparative analysis of apicomplexan hemoprotozoa. PLoS Pathog 3: 1401–1413. 17953480 10.1371/journal.ppat.0030148 PMC2034396

[24] Freeman JM, Kappmeyer LS, Ueti MW, McElwain TF, Baszler TV, et al (2010) A *Babesia bovis* gene syntenic to *Theileria parva* p67 is expressed in blood and tick stage parasites. Vet Parasitol 173: 211–218. 20638797 10.1016/j.vetpar.2010.06.024

[25] Machado RZ, Valadao CA, Melo WR, Alessi AC (1994) Isolation of *Babesia bigemina* and *Babesia bovis* merozoites by ammonium chloride lysis of infected erythrocytes. Braz J Med Biol Res 27: 2591–2598. 7549981

[26] Larkin MA, Blackshields G, Brown NP, Chenna R, McGettigan PA, et al (2007) Clustal W and Clustal X version 2.0. Bioinformatics 23: 2947–2948. 17846036 10.1093/bioinformatics/btm404

[27] Davies DH, Liang X, Hernandez JE, Randall A, Hirst S, et al (2005) Profiling the humoral immune response to infection by using proteome microarrays: high-throughput vaccine and diagnostic antigen discovery. Proc Natl Acad Sci U S A 102: 547–552. 15647345 10.1073/pnas.0408782102 PMC545576

[28] Doolan DL, Mu Y, Unal B, Sundaresh S, Hirst S, et al (2008) Profiling humoral immune responses to P. falciparum infection with protein microarrays. Proteomics 8: 4680–4694. 18937256 10.1002/pmic.200800194 PMC3021802

[29] Vigil A, Ortega R, Jain A, Nakajima-Sasaki R, Tan X, et al (2010) Identification of the feline humoral immune response to *Bartonella henselae* infection by protein microarray. PLoS One 5: e11447. 20625509 10.1371/journal.pone.0011447 PMC2897887

[30] Birkenheuer AJ, Levy MG, Breitschwerdt EB (2003) Development and evaluation of a seminested PCR for detection and differentiation of *Babesia gibsoni* (Asian genotype) and *B. canis* DNA in canine blood samples. J Clin Microbiol 41: 4172–4177. 12958243 10.1128/JCM.41.9.4172-4177.2003 PMC193857

[31] Ronquist F, Teslenko M, van der Mark P, Ayres DL, Darling A, et al (2012) MrBayes 3.2: efficient Bayesian phylogenetic inference and model choice across a large model space. Syst Biol 61: 539–542. 22357727 10.1093/sysbio/sys029 PMC3329765

[32] Susta L, Torres-Velez F, Zhang J, Brown C (2009) An in situ hybridization and immunohistochemical study of cytauxzoonosis in domestic cats. Vet Pathol 46: 1197–1204. 19605894 10.1354/vp.08-VP-0132-B-FL

[33] Debierre-Grockiego F, Schwarz RT (2010) Immunological reactions in response to apicomplexan glycosylphosphatidylinositols. Glycobiology 20: 801–811. 20378610 10.1093/glycob/cwq038

[34] Wagner JE, Ferris DH, Kier AB, Wightman SR, Maring E, et al (1980) Experimentally induced cytauxzoonosis-like disease in domestic cats. Vet Parasitol 6: 305–3111.

