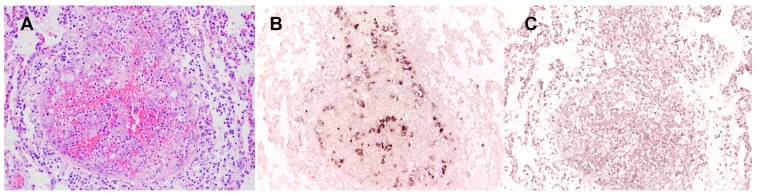# Correction: A Novel Candidate Vaccine for Cytauxzoonosis Inferred from Comparative Apicomplexan Genomics

**DOI:** 10.1371/annotation/943b121e-343b-4df1-a06b-7f8a205a057d

**Published:** 2013-10-23

**Authors:** Jaime L. Tarigo, Elizabeth H. Scholl, David McK. Bird, Corrie C. Brown, Leah A. Cohn, Gregg A. Dean, Michael G. Levy, Denise L. Doolan, Angela Trieu, Shila K. Nordone, Philip L. Felgner, Adam Vigil, Adam J. Birkenheuer

In the PDF version of the article, A duplicate version of Figure 3 appeared instead of Figure 6. The correct version of Figure 6 is: 

**Figure:**