# Supplementary figures and images for: A Novel Candidate Vaccine for Cytauxzoonosis Inferred from Comparative Apicomplexan Genomics
Source: PLoS One. 2013 Aug 20;8(8):e71233. doi: 10.1371/journal.pone.0071233 (PMC3748084; doi:10.1371/journal.pone.0071233)

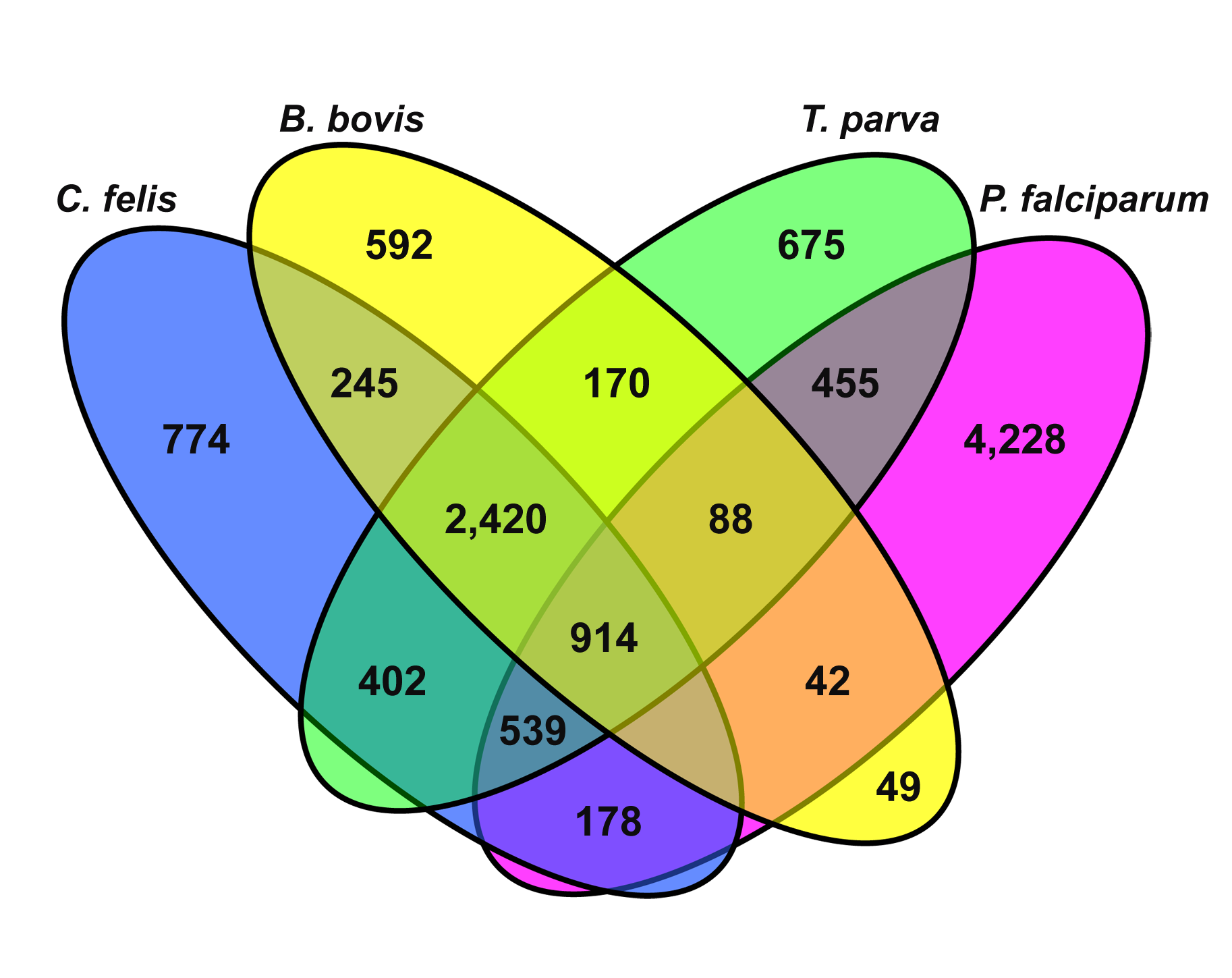

Supplement: Figure S1 — Four way Venn Diagram: Protein coding genes of Cytauxzoon felis and related apicomplexan parasites. (TIF) [file pone.0071233.s001.tif]

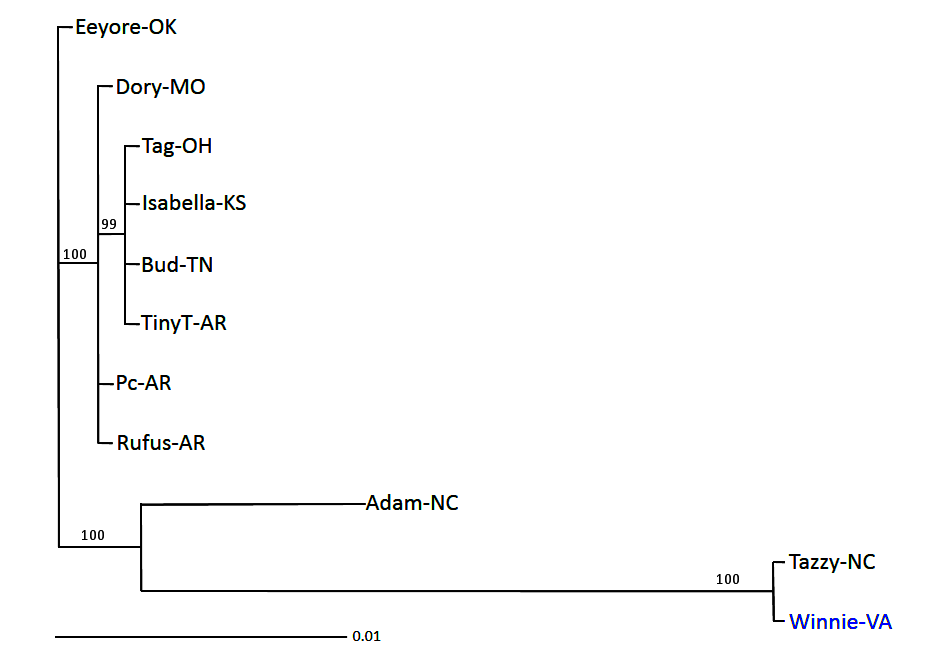

Supplement: Figure S5 — Phylogenetic reconstruction of cf76 across 11 isolates. Posterior probability of clades is indicated on branches. Sample names are based on the name of the individual animal and the state from which the sample was taken (AR - Arkansas, KS - Kansas, MO - Missouri, NC - North Carolina, OK - Oklahoma, OH - Ohio, TN - Tennessee, VA - Virginia). Scale bar indicates expected changes per site. Full genome sequence is derived from Winnie-VA. (TIF) [file pone.0071233.s005.tif]
