# Supplementary material for: A Novel Candidate Vaccine for Cytauxzoonosis Inferred from Comparative Apicomplexan Genomics
Source: PLoS One. 2013 Aug 20;8(8):e71233. doi: 10.1371/journal.pone.0071233 (PMC3748084; doi:10.1371/journal.pone.0071233)
Supplement: Figure S2 — Nucleotide sequence alignment of syntenic genes C. felis cf76-1 (isolate from the C. felis genome sequence), cf76-2 (the most common sequence among eleven geographically diverse C. felis isolates), T. parva p67 (GenBank U40703.1), T. annulata SPAG-1 (GenBank M63017.1), and B. bovis BOV57 (GenBank FJ805276.1). (DOC) [file pone.0071233.s002.doc]

C.felis_cf76-1 ------------------------------------------------------------

C.felis_cf76-2 ------------------------------------------------------------

T.parva_p67 ------------------------------------------------------------

T.annulata_SPAG-1 GTTTTAAAAAGGAGTAACATTGGAATTTAAATTTCATTTTCCAACACTCAACGATGAATA

B.bovis_BOV57 ------------------------------------------------------------

C.felis_cf76-1 ------------------------------------------------------------

C.felis_cf76-2 ------------------------------------------------------------

T.parva_p67 ------------------------------------------------------------

T.annulata_SPAG-1 TTATACACTTTCTGTTGACCATTCCGGCTATTTTTGTATCTGGAGCGGACAAGATGCCTG

B.bovis_BOV57 ------------------------------------------------------------

C.felis_cf76-1 ------------------------------------------------------------

C.felis_cf76-2 ------------------------------------------------------------

T.parva_p67 ------------------------------------------------------------

T.annulata_SPAG-1 CGGGAGAAAGTTCTAGAACCTCTAAACCCAGTCCCCTAGTAACCCTAGAATCGGCGGTAA

B.bovis_BOV57 ------------------------------------------------------------

C.felis_cf76-1 ------------------------------------------------------------

C.felis_cf76-2 ------------------------------------------------------------

T.parva_p67 ------------------------------------------------------------

T.annulata_SPAG-1 CACAACCTTCAAAGGACCCATTCAAGACAATTAGTGCCTTGTCAAAAGCAACAAAAGTAT

B.bovis_BOV57 ------------------------------------------------------------

C.felis_cf76-1 ------------------------------------------------------------

C.felis_cf76-2 ------------------------------------------------------------

T.parva_p67 ------------------------------------------------------------

T.annulata_SPAG-1 GGAAGTCAGCGGTATCAGTATCAGGTGACTCTAAGACTGTACCTACTCCAGTTTCGGAAC

B.bovis_BOV57 ------------------------------------------------------------

C.felis_cf76-1 ------------------------------------------------------------

C.felis_cf76-2 ------------------------------------------------------------

T.parva_p67 ------------------------------------------------------------

T.annulata_SPAG-1 CAATGATCACTCGATCTTTTCAAGAACCAGTATCTCAAGAACTTGAATTCCAATCAGATA

B.bovis_BOV57 ------------------------------------------------------------

C.felis_cf76-1 ------------------------------------------------------------

C.felis_cf76-2 ------------------------------------------------------------

T.parva_p67 ------------------------------------------------------------

T.annulata_SPAG-1 CTGAAATTAATGAGTCAGGATCCGGTTCAGATGAGGATGAGGATGACGATGACGATGAGG

B.bovis_BOV57 ------------------------------------------------------------

C.felis_cf76-1 ------------------------------------------------------------

C.felis_cf76-2 ------------------------------------------------------------

T.parva_p67 ------------------------------------------------------------

T.annulata_SPAG-1 AGGAAGAAGAAGACGATAAATCTACCTCATCTAAAAACGGAAAAGGCAGCCCAAAAGCTC

B.bovis_BOV57 ------------------------------------------------------------

C.felis_cf76-1 ------------------------------------------------------------

C.felis_cf76-2 ------------------------------------------------------------

T.parva_p67 ------ATGCGACACTGAACGATGCAAATAACTCAGTTTTTGCTGATC--ATTCCGGTGT

T.annulata_SPAG-1 AGCCTGGAGTATCTTCAAGCAGTACATCCTCAGCAAGTCCAACATCTCCAACTACAACAT

B.bovis_BOV57 ------------------------------------------------------------

C.felis_cf76-1 ------------------------------------------------------------

C.felis_cf76-2 ------------------------------ATGATGAAATTTTTATTAATGTTTGTGGTG

T.parva_p67 TTTTTGTATCCGCAG-GGGACAAAATGCCCACGGAGGAACAACCATTTCCTTCTAGGCTT

T.annulata_SPAG-1 TATCACAAACTGGATTGGGACCAAGTGGTTCCC-ACGCTCAACAAGATCCCGGTGTAGGT

B.bovis_BOV57 ------------------------------------------------------------

C.felis_cf76-1 ------------------------------CCTGTAGCTGCTGCACAACCACAACCAGCT

C.felis_cf76-2 CCTTTGATGACATTGGCTGTAGATCCAGAACCTGTAGCTGCTGCACAACCACAACCAGCT

T.parva_p67 AGTCCCCTAGTAA---CCTTGGAATCAGCCATAACACAACCTACCCCTGTGTACACAATG

T.annulata_SPAG-1 GTTCCAGGAGTTGGTGTTCCAGGAGTAGGTGTTCCAGGAGTAGGTGTTCCAGGAGTAGGT

B.bovis_BOV57 ------------------------------------------------------------

C.felis_cf76-1 GTGACTGGTGTACAACAGGTGATGCCTACTAATGCACCTGTAGTGGTTACTGGACAACCT

C.felis_cf76-2 GTGACTGGTGTACAACAGGTGATGCCTACTAATGCACCTGTAGTGGTTACTGGACAACCT

T.parva_p67 AGGTCAGTTGGTAACGTGGCAAAGGCAGCAAAGGTATG-GAAGTCAGCA--GTATCATCA

T.annulata_SPAG-1 GTTCCAGGTGTAGGTGTGCCAGGTGTAGGAGGTGTTCCCGGAGTTGGCGTTGCACCAGGG

B.bovis_BOV57 ------------------------------------------------------------

C.felis_cf76-1 GCGAGTACACCAGCAGTTGTTAATCAACAATCTATAC-CACAGGCAC--CTAATACTCCA

C.felis_cf76-2 GCGAGTACACCAGCAGTTGTTAATCAACAATCTATAC-CACAGGCAC--CTAATACTCCA

T.parva_p67 CCAGATGTCTCTACTACTATTCCCACTCCAGTT-------TCGGAA--GAAAATATCACA

T.annulata_SPAG-1 GTAGGTGTTCCAGGAGTTGGTGTTGCACCAGGTGTAGGTGTTGGAGCTGATAGTAGTGGA

B.bovis_BOV57 ------------------------------------------------------------

C.felis_cf76-1 GTAGTTGCTACTGGTCAAGATGCAACGGATGTTAGAAGTATAACGAATGTCACAGCACCT

C.felis_cf76-2 GTAGTTGCTACTGGTCAAGATGCAACGGATGTTAGAAGTATAACGAATGTCACAGCACCT

T.parva_p67 TCAACT---ATTCATACACAAACGAAAGAAGTTCCTGCTG---CAAGCGGCTCAGATTC-

T.annulata_SPAG-1 TTGCCTGGAAGTGGTGGTCTTGGAGCAGGAGCAAAGGCTG---GGAAAGGTCAAGGATCT

B.bovis_BOV57 ------------------------------------------------------------

C.felis_cf76-1 ACAGTTCAGCAACCATTA----CCAGTACAACCACCGCAACAGATATTACAAGTACCGAT

C.felis_cf76-2 ACAGTTCAGCAACCATTA----CCAGTACAACCACCGCAACAGATATTACAAGTACCGAT

T.parva_p67 -ATCCACTGTAACAAAT-----TTGGTACAAACACAATCCCAAGTTCAGGATAATGTAAA

T.annulata_SPAG-1 GGTCTACAGGGACCAGGAGGTGTTGGAGTAGTACCTGGTGTAGGTGTAGCAGCTTCTTCT

B.bovis_BOV57 ------------------------------------------------------------

C.felis_cf76-1 TGTACAACAAGCGG--TACCAGCGGTCCAACCTTCAGCTGGTATTGAAGTTAA---GGAT

C.felis_cf76-2 TGTACAACAAGCGG--TACCAGCGGTCCAACC---AGCTGGTATTGAAGTTAAAAAGGAT

T.parva_p67 GCAACAGCAAGATA--CTAAGGGAAACAAAACAGATTCCGAAGAAGAAAATGAAGAT--A

T.annulata_SPAG-1 TCTTCACCAGGAAAACCTCCAGGAGTAGGAGCAGGAGTTATGCCTGGAGTTGGTGTACGA

B.bovis_BOV57 ------------------------------------------------------------

C.felis_cf76-1 AATACATCAACAGCACCAGCTAATCTAGAAAATGCCATTACTGTACCTTCTGTAGTTCCA

C.felis_cf76-2 AATACATCAACAGCACCAGCTAATCTAGAAAATACCACTACTGTACCTTCTGTAGTGCCT

T.parva_p67 GCACCGTTAGTACAGATGTCTCTCCGACCATTCCTACTCCA-GTATCAGAAGAAA--TTA

T.annulata_SPAG-1 GCACAAGGAGGAGTAATAATTGGTGCGCCAGGAGTAGCAGGTGTGCCAGGAGGAAAGCCA

B.bovis_BOV57 ------------------------------------------------------------

C.felis_cf76-1 TCTGTAGGTAGTCCATCAGTTCC-------AACAACAGTTCCTCCAACAGGAGTGA----

C.felis_cf76-2 GCGGTAGGTAGTCCATCAGTTAC-------AACAACAGTTCCTCTACCAGCAGTGG----

T.parva_p67 TC-ACA-CCAACTCTTCAAGCAC---AAACGAAAGAAGAAGTTCCTCCTGCAGACC----

T.annulata_SPAG-1 GG-ACAACCAGTATCTCAAGAACTTGAACTGAAATCAGACACTGAAATTAATGAGTCAGG

B.bovis_BOV57 ------------------------------------------------------------

C.felis_cf76-1 CCACAACTCAAGATAGAACAA--ATGTTCCAACTGCCATGGAAG------------GTTC

C.felis_cf76-2 CAACAACTCAGGATAGAACAA--ATGTTCCCACTGTTGTAGAAG------------CTTC

T.parva_p67 TCTCAGATCAAGTTCCGTCAA--ACGGCTCAGACTCCGAAGAAGAAGATAATAAATCCAC

T.annulata_SPAG-1 TTCCAGTTCAGAAGGGGAAGACGATGACGATGAAGAAGAGGAAGAAGAAAATAAATCTAC

B.bovis_BOV57 ------------------------------------------------------------

C.felis_cf76-1 ACCACCTGAGGTTAAAACCACTGT---TCCAGTAAGAGTTGCATCCGAGGTCCAATCCAC

C.felis_cf76-2 TCCACCTGAGGTTA---------------------------CATCC----------TCAC

T.parva_p67 CTCATCTAAAGATG-AAAAGGAAC--TCAAAAAAACTCTACAACCCGGAAAAACATCCAC

T.annulata_SPAG-1 CTCATCTAAAGGAGCAGGAGGAAAGGCTGGAAAAGGTCAAGGATCTGTA---TCACCAGG

B.bovis_BOV57 ------------------------------------------------------------

C.felis_cf76-1 GTCTGTTCCTCTTGCAG---AGAGCTCTCCGCCCGAGGTCAGTCCTGCCGTCAGCTCTTC

C.felis_cf76-2 A-----TTCTCCTGCAG---AGAGCTCT------------------------------TC

T.parva_p67 AGGTGAAACTACATCGGGCCAAG-ATCTTAATTCAAAACAACAGCAAACTGGT---GTAT

T.annulata_SPAG-1 AGGAGGATCCTCAGCAAGTCAAACATCTCCAACTACAACACCA-CAATCTGGCTTGGCAT

B.bovis_BOV57 ----ATGGCATTTGCAAAGTTGTCTATTTTGTTTACGTTTTTATTAGTACATT-TAGTGT

C.felis_cf76-1 CAATTACCTATCCCAAATGGGTAGAGCCACTCCCGGAGATCGTGGCGGAAGTATAGTCAC

C.felis_cf76-2 TAATTTGTTATCCCAACTGGGTAGAGCCACTCCCGGAGATCGTGGCGGAAGTATAGCCAC

T.parva_p67 CAGATCTAGCCAGTGGATCACACTCTTCTGGACTTACAGTACCTGGAGTAGGAGTTCCAG

T.annulata_SPAG-1 CAAGTGGTTCTCATGCTCAACAAAGTCCTCAAC--AAGATCCAGCGCCTAGTAAACCTAG

B.bovis_BOV57 CTACTAATGCCTTCGATTTGGGTGAATGGTCAC--ATGATGCGCATGACACCCATGATAT

C.felis_cf76-1 -TGGACCTCAAGTGGA---------------ACCCGTAGCTGATCTCAGGGAAGCGGAGG

C.felis_cf76-2 -TGGACCTCAAGTGGAGACCTCAAGTGTAAAACCCGCAGCTGATCTCAGGGAAGCGGAGG

T.parva_p67 GTGCAGTTTCTCCCCAAGGAGGTCAATCTCTAGCTTCGAATACATCTAGAGAAGGTCAGA

T.annulata_SPAG-1 -TGGAGGAGGTGTGCCAGGAGTTGGAGTTCCTGGTGTTGGCGTTCCCGGTGTTGG-----

B.bovis_BOV57 -CAAAGCTCATCATGAA---------TCCGCGGCTGATGCGACTCCTGGTGCGGCTGCAC

C.felis_cf76-1 GACAAGTAAATAGAGGAGGCGCCACGCCTAGTGGAATGG---ATGGTAGAAATGTAA--C

C.felis_cf76-2 GACAACTAAATAGAGAAGGCACCACGCCTAGTGGAAGAGCTGACGGCAGGAATGTAA--C

T.parva_p67 CGCAACATCAACAGGCAAGAGATGGAGATGGTAGAGTTATTGAGCCTAAAATTGGATTAC

T.annulata_SPAG-1 ---AGTACCAGGAG-TAGGAGTTGCGCCGGGAGTTGGTGTTGTACCTGGAGTAGGGG---

B.bovis_BOV57 AGACGCTCAGCGAATCTGAGGAAATGGA-GAAGGCGCTGAAGGCACTAGAGGAGGAGA--

C.felis_cf76-1 ATCGGGTGGAATATTGGAT-GGAAATATTATTA-CGGATGGTTTACCGATTGGTATAAAT

C.felis_cf76-2 CTTGGGTGGAATGTTGGAT-GGGAATATTATTA-CGGATGGTTTACCGATTGGTACAAAT

T.parva_p67 CCGGATCTACATCTGCGCC-AGTACCAACACGACCACCCGGTTCATCTACAGATACAAGA

T.annulata_SPAG-1 GTGCAACAACTTCTTCATC-ATCAACAACTTCAACTTCAACTTCAACTACTACTACTACT

B.bovis_BOV57 CTAAACTGGAAAATAAGCCTAATGACACCCCTA-CTCCTGTTCCTGTGACTCCTAGCGCG

C.felis_cf76-1 ATA--TCGGCTAGTAGTGAGAGTGATATAGCAACATCAAGG-ATATTATTAGGTATTG--

C.felis_cf76-2 GTA--TCAGCTAGTAGTGAGGGTGATATAACAACATCTAGG-ATATTATTAGGTATTG--

T.parva_p67 CCAGCCTCATCAGGA---CCTTCAGCACCAGGTGGTCCGGGTTCATCTTCAAGAAGTGGA

T.annulata_SPAG-1 ACAACTTCATCAGGAAAACCTTCAGACCAAGGAAGCCATGGTACTTCTCCAAGAAATGCA

B.bovis_BOV57 GAAGAGAAACAAGATGCTCCTGTGGAGGAAAATAAAAAGGTTGACCAACCGAAAATTGAA

C.felis_cf76-1 GTAATGAGATGTCATTGAT--AGTAGATGAGATATTAGTAAAATTAGAAGAATTAAAGGT

C.felis_cf76-2 GTAATGAGATGTCATTGAT--AGTAGATGAGATATTAGTAAAATTAGAAGAATTGAAAGT

T.parva_p67 GGCACCAGATCA-ACTGATTCAGTAACAAGGCCGGTACCATCACCAGGAGCGCCCGGAAT

T.annulata_SPAG-1 GTAACCAGACAA-ACTGACTCAATATCAGGACCCATACCATCACCAGGAGATCCAAGAGC

B.bovis_BOV57 ATACCTGCACTACATCCTC---CTGACTCGCCTCTTCACACCG--AGAAGGAT----GAT

C.felis_cf76-1 A-TTAGAGGATAAGAAATT--------------------------GGTTGGTAATACACA

C.felis_cf76-2 A-TTAGAGGATAAGAAATT--------------------------GGTTGGTAATACACA

T.parva_p67 AATTATTAGAGAATTAGGTTTGTTTTTTTGAGTATATGGGTTTTAGGTAATAGGGCAATG

T.annulata_SPAG-1 AATTACTGGACAAATGG-----------------------------GTGAAGGAGAAAGG

B.bovis_BOV57 GCTTTGGACATAACTAC------------------------------AGCGCCATTTACG

C.felis_cf76-1 GAAATTAGAGAGTTTAAGAGAGAGTATAATAACAGAATATCAAAAATTTATACAAGAGAT

C.felis_cf76-2 GAAATTAGAGAGTTTAAGAGAGAGTATAATAACAGAATATCAAAAATTTATACAAGAGAT

T.parva_p67 GATATTGTACAGTTTTTAGGAAGATTTAA-ACCAGAACCAAGGACATATGAAGGGGACAG

T.annulata_SPAG-1 TTTGCTGTACAGTTCCTGGGAGATTTTAA-ACCAAAACCAAGGAGATATGAAGGACAAGG

B.bovis_BOV57 TTGGTAGAAGACCCTGCGAGTCACGAGAACGAGCTAACATCAGAAATTCCCCAAAGTCCT

C.felis_cf76-1 AACAGAGATAGAAAATTCAGATGAGAATACAAAAATGGATGGTATACAATCATCAGATAT

C.felis_cf76-2 AACAGAGATAGAAAATTCAGATGAGAATACAAAAATGGATGGTATACAATCATCAGATAT

T.parva_p67 AACAAATGTAGCAGAACTAAAAAAATTCCTATTTGAAGAACTTGAATCTTTGGTAAACAC

T.annulata_SPAG-1 AACAGATGCAGTAAAACTAAAACAATTCATTTTCGAAGAGGTCAAATCGCTGGTGCAAAC

B.bovis_BOV57 GCGGATGATACTAATGTCAACGC------TGGTAATGAGGATTCTATTATAACTGATACT

C.felis_cf76-1 AGCA-CAAACATTAAGATATAAATATGATGCATCAGTAAAAAATATAATGGCAAATGTAA

C.felis_cf76-2 AGCA-CAAACATTAAGATATAAATATGATGCATCAGTAAAAAATATAATGGCAAATGTAA

T.parva_p67 TCTAATAGAATTGAAATTAGCAATTGCAAACGACTTTGTTGAAATCACTGATGGTTTGAG

T.annulata_SPAG-1 CTTAATAAACCTTAAATTAGCAATTGCAAACGACTTTGTTGAAATCAGTGAAAAGTTGAA

B.bovis_BOV57 ACTCCCATAGCTAAGAGCATGCGTCTGA-ACACTGTCACTAAAATTGACGAGACCATTGA

C.felis_cf76-1 TGAAAATATTAAATA-CAAAAGGTAAATATGATGGTGCTATATTAGCATATAATTATATT

C.felis_cf76-2 TGAAAATATTAAATA-CAAAAGGTAAATATGATGGTGCTATATTAGCATATAATTATATT

T.parva_p67 AAAGAATACTAAAGATCATGAAGCCAGATTGAAGTTGCTA-AAAGGTGTAGAATTCACTA

T.annulata_SPAG-1 AAAGAAAAATCAAAATTACGTACCGAAATTAAAGTTGTTA-AAAGGAGAACAATTTGACA

B.bovis_BOV57 AAAGCTTAACCATCGTCTCCAGACGTTTTTGGAGTCTGTA-----GCGT-CATCTGCTCA

C.felis_cf76-1 AAAGATAAAGTACAATCAAT-TA--AAAATGGTATAA------AAAATCCATCATCTGAA

C.felis_cf76-2 AAAGATAAAGTACAATCAAT-TA--AAAATGGTATAA------AAAATCCATCATCTGAA

T.parva_p67 AGAGGAAAAGTGTCGCCAACGTAGTAAAGGGGTTTAGTTCTTTGTACTGTGTGCTTTTAA

T.annulata_SPAG-1 CCAAACAGAAGGTAGCCAACGTACTAAAAGGGTTCAATTCTCTGTACTTCGTATTTTTTA

B.bovis_BOV57 CGACCTTACGTATTATCAGTCTTTGTTAGATACTGCCTATGATATATTCTGCAGGGAGAT

C.felis_cf76-1 T--ATCTTA-AACTTATAAGAGATATAGATTTTAGTGCTGA--TAATATAATAGACCCAA

C.felis_cf76-2 T--ATCTTA-AACTTATAAGAGATATAGATTTTAGTGCTGA--TAATATAATAGACCCAA

T.parva_p67 TGAATATGA-ACGGCATCAAAGAAAAAAAGAGAGAATCTGAAGTAGCAGATGGCATTTGG

T.annulata_SPAG-1 TGAACCTTA-ACCTAGCGAAAGAAGTTAACAAACCGGAAGAATTGGCAGAATTTCTTTGG

B.bovis_BOV57 TAACGGTGACATGTCGCCGATGGGAAGTGGTGGGCAGCTGGATAAGGATGGTA-ATGGAG

C.felis_cf76-1 TGATTAATAATGA----AGAAAAAGTTGGTATACA-ATTAAAAGATGCTAAATCA--AAA

C.felis_cf76-2 TGATTAATAATGA----AGAAAAAGTTGGTATACA-ATTAAAAGATGCTAAATCA--AAA

T.parva_p67 AAACTGTCTACACTCCCAGATAAAGTAGCAAATGA-ACTTTTGTTAGCTATGGAA--AAG

T.annulata_SPAG-1 AAACTAAATACAATCCCAGATAAAGTAGGAAGAGA-ATTTGAGTTAGCAATAGAA--AAA

B.bovis_BOV57 TGACC-CTTATGATTAGTGCTGAGATGTCCAGTGCCATTCGTCGTAGTTTTGATACCAAG

C.felis_cf76-1 ATATTTGGTTTATTATCTAATAATACAAATAATAATATTACTTA-TGATCTTAAAAAAAA

C.felis_cf76-2 ATATTTGGTTTATTATCTAATAATACAAATAATAATATTACTTA-TGATCTTAAAAAAAA

T.parva_p67 ATCGTGGTCCCACCAAAAACCCCTG-AACTAGAAGAAGCATTTA-AGGCAATTGAGTTTG

T.annulata_SPAG-1 ACTAAAGGTTCAGAGAAAAAGAAGG-AATTAGAAGAAGCATTTA-ATTCAATAGGGTTAG

B.bovis_BOV57 GTGGAGGTTTTAGAGTTGGCTGCTT---CTGAGGTGGCCAGTCAGAAGTCCAAGGAAGTG

C.felis_cf76-1 AATTATTGAACATTTTAATTCATTACAAGAAGAACATTCAATAGC-TAATTCATT-AATT

C.felis_cf76-2 AATTATTGAACATTTTAATTCATTACAAGAAGAACATTCAATAGC-TAATTCATT-AATT

T.parva_p67 G-TTTCAAAATAGCATACTACGCAACCAAAGACATCCTCTCAAGTATAGAAAACACAGTT

T.annulata_SPAG-1 G-TTTCAAAATAGCACAGTACGCAACAAATGACATCCTCTCAAGTATAACAAATTCAGTC

B.bovis_BOV57 GGTGCCCAAACTATTCACGACGCCCTTACCGTAGGCCTCAGAACTGTTAGAGATA----C

C.felis_cf76-1 AATGGTGCTAAAAAATTTTCTAATAAACTTGAACATCTAACTAATAAACTTAAAATCTCA

C.felis_cf76-2 AATGGTGCTAAAAAATTTTCTAATAAACTTGAACATCTAACTAATAAACTTAAAATCTCA

T.parva_p67 CACAACTTGATGCACGCCAAAAATTATGAAGAGAATTTTATTGCTCAAGTAAGAA----A

T.annulata_SPAG-1 TACTCCCTGATAAAACTAAAGAATTTTGGAGATGATTTTGTTACCGAAGTAAGAA----A

B.bovis_BOV57 CATAACCTCTCCGGGCATGACAATTCACACGACAAGC-----AATGACATGAAGAACATG

C.felis_cf76-1 ATTTCTAAATATGTTGCTACTGCTGATGAATCCAATACTATCAA-ATTCATACATCAAGC

C.felis_cf76-2 ATTTCTAAATATGTTGCTACTGCTGATGAATCCAATACTATCAA-ATTCATACATCAAGC

T.parva_p67 CTCTCTAAGGATGGTGCCACACCAGATGAACTTGACTGAATCGTCGTTTGTAATTAAAAT

T.annulata_SPAG-1 GTCACTGCAAATGGTTCCACACCAAAAGAACCTAAACGGATCAGCATTTATAGTCAAAAT

B.bovis_BOV57 ACTGCTATTGTAGCTGACATGTCCAAAGGCCTC---TTGGCCGACATAATTAAGTGGACA

C.felis_cf76-1 ATCAAATGCACTCG---AAAAAACTAATAATACACAAATTATAATGAATACAACCAATGA

C.felis_cf76-2 ATCAAATGCACTCG---AAAAAACTAATAATACACAAATTATAATGAATACAACCAATGA

T.parva_p67 CTCAGACATGATGCGC-AGAAGAGGAACAGCAAGTCAGGACCAACCAGCAGGAGCTGGGT

T.annulata_SPAG-1 CTCAGAAATAATCAAC-AAAAAAGGAACAGAAGATCAGGATCAAACATCAGGAAGTGGGT

B.bovis_BOV57 CTAAAGGAAGATGTTTTAAAGAAGCGACTGTTCGACAAGATAGTTGAGCGTGA-CAATTT

C.felis_cf76-1 TAGTAATGCTGTCAAATCTACATCCGATGTCCAATCCATGT-CTGTTCCTCTTGCAGAGA

C.felis_cf76-2 TAGTAATGCTGTCAAATCTACATCCGATGTCCAATCCATGT-CTGTTCCTCTTGCAGAGA

T.parva_p67 CCGCAGTAACACCAGGACGAGGATCATCAGGTACGGGACGAGCAGCAGGAACCGGAGGAG

T.annulata_SPAG-1 C------------------------------------------AAAAGGAACAGAAGGAG

B.bovis_BOV57 C---------ATCAAGACGTCGCCTGACG--------------AGCTCCGTATGGAGGCA

C.felis_cf76-1 GCTCTTCTAATTTATTATCCCAAATGGGTAGAGCCACTCCCAGAGATCGTGGCGG--TAA

C.felis_cf76-2 GCTCTTCTAATTTATTATCCCAAATGGGTAGAGCCACTCCCAGAGATCGTGGCGG--TAA

T.parva_p67 GATCACTGAGGGGATTAGACTTAAGTGAAGAAGAAGTTAAGAAAATCTTGGATGAAATAG

T.annulata_SPAG-1 GATCACTAAGGGGGCAAGATTTGACAGAAGAAGAAGTTTTGAAAGTTCTGGATGAACTAG

B.bovis_BOV57 -TTCACTCACGCAATCCG-TGAATTGGCTGGAGAATTTCACAATGCACAGAAAGA---GA

C.felis_cf76-1 TGAAGGTAGTGATGGAATGAAGAGTAGTACT--GGACCTCAAGTGGAACCCGCAGCTGAT

C.felis_cf76-2 TGAAGGTAGTGATGGAATGAAGAGTAGTACT--GGACCTCAAGTGGAACCCGCAGCTGAT

T.parva_p67 TGAAAGATCCAAGCGACGGAGAACTTGGACTCGGAGACTTAAGTGACCCAAGTGGAAGAT

T.annulata_SPAG-1 TGAAGGATGTAAGCGAAGAACATGTTGGAATAGGAGATTTAAGTGACCCAAGTAGCAGAA

B.bovis_BOV57 AGACTGGTGCTATCGAAAAGCAGAACGGTTTC-AAGGAGTACATGGACGAAATGCGTGAG

C.felis_cf76-1 CTCA----GGGAAGCGGAGG------GGGAAGTAAATAAAGAAGCTGACGGCAGGAATGT

C.felis_cf76-2 CTCA----GGGAAGCGGAGG------GGGAAGTAAATAAAGAAGCTGACGGCAGGAATGT

T.parva_p67 CATC---CGGAAGACAGCCCTCACTCGGACCTTCACTTGGAATAACTGATGGAGAAGCAG

T.annulata_SPAG-1 CACCAAATGCAAAACCAGCCGAACTTGGACCTTCACTAGTGATACAAAATGTACCGTCAG

B.bovis_BOV57 GACA----TCAACACCATTCAGAGGCTTATTGACACATACTTTGCC-ACTGTGCACAAAG

C.felis_cf76-1 AACCTCGGGTGGAGAAGC-TGACGGCAGGAATGTAACC-TCAGGTGGAAAGACTTCAAGT

C.felis_cf76-2 AACCTCGGGTGGAGAAGC-TGACGGCAGGAATGTAACC-TCAGGTGGAAAGACTTCAAGT

T.parva_p67 GACCCACAATAGTATCTC-CAACAGGGCCCACAATAGCAGCTGGAGGAG-AACAACCACC

T.annulata_SPAG-1 ACCCCTCAAAAGTGACAC-CAACACAGCCTTCAAATTTGCCACAAGTACCAACAACAGGG

B.bovis_BOV57 GTCATGCTAAGGCTATTCTCTACGAGGCAAGCAAGGAGCTCA---GAAAAGATGGTAATG

C.felis_cf76-1 TTAGAAGACAATAC--ATGGAATTACGGTGGAATAAATACAGAAAATACCAA-AGCTAAA

C.felis_cf76-2 TTAGAAGACAATAC--ATGGAATTACGGTGGAATAAATACAGAAAATACCAA-AGCTAAA

T.parva_p67 TTCAGCTCCTA-----ATGGAACCGCAAAGGGACCAGCAGGAA-------------CACA

T.annulata_SPAG-1 CCGGGGAACGGGACGGATGGAACAACAACAGGACCAGGTGGAAACGGGGAAGGAGGCAAA

B.bovis_BOV57 CCGAGAGCCAAT-----TGCGCTTACGTGTGGCTGAGCAG-----------------AAG

C.felis_cf76-1 GGTAATTTAAAAGGTAAAGAAGAAGGTGAATTGAAGTTGGTAGATGATGAAGATGAGGAA

C.felis_cf76-2 GGTAATTTAAAAGGTAAAGAAGAAGGTGAATTGAAGTTGGTAGATGATGAAGATGAGGAA

T.parva_p67 GCCT--GAGGGAGGAGAGAAGAAAGAAGGATTGATACAGAAGCTCAAGAAAAAATTCCTG

T.annulata_SPAG-1 GATTTGAAGGAAGGAGAAAAGAAAGAAGGATTATTTCAAAAGATCAAAAACAAACTCTTG

B.bovis_BOV57 GTTCATCAAGAGGAACTTAAAAAGGCAGAACCAAAGCAGGAGGATACGGGATGTCCGTAT

C.felis_cf76-1 GAAGCTGTAAAGGATGGATTCAATCACATTAAAATAATCGCTAC-ATTATTGTTATCATT

C.felis_cf76-2 GAAGCTGTAAAGGATGGATTCAATCACATTAAAATAATCGCTAC-ATTATTGTTATCATT

T.parva_p67 GGGTCTGGATTCGAAGTCGCGAGTCTTATGATACCAATGGCGACGATAATTATCAGCATC

T.annulata_SPAG-1 GGCTCAGGATTCGAAGTCGCAAGTATTATTATACCAATGACAACAATCATATTCAGCATA

B.bovis_BOV57 CCAACCGAACCTCA----------------------------------------------

C.felis_cf76-1 AAC-ACTAGTTTAA-------------------------------------------

C.felis_cf76-2 AAC-ACTAGTT----------------------------------------------

T.parva_p67 GTCCACTAACAATAACTC---------------------------------------

T.annulata_SPAG-1 GTCCACTAAAACTAAAAACACAACTAACCACACTAATTTATAATATACAAAAAAAAA

B.bovis_BOV57 ---------------------------------------------------------
