# Supplementary material for: A Novel Candidate Vaccine for Cytauxzoonosis Inferred from Comparative Apicomplexan Genomics
Source: PLoS One. 2013 Aug 20;8(8):e71233. doi: 10.1371/journal.pone.0071233 (PMC3748084; doi:10.1371/journal.pone.0071233)
Supplement: Figure S3 — Amino acid sequence alignment of syntenic genes C. felis cf76-1 (isolate from the C. felis genome sequence), cf76-2 (the most common sequence among eleven geographically diverse C. felis isolates), T. parva p67 (GenBank AAB06703.1), T. annulata SPAG-1 (GenBank AAA30134.1), and B. bovis BOV57 (GenBank ACY08791.1) (shading: black- identical amino acids, grey- similar amino acids). (DOC) [file pone.0071233.s003.doc]

C.felis_cf76-1 MMKFLLMFVVPLMTLAVDPEPVAAAQPQPAVTG---VQQVMPTNAPVVVTGQPASTPAVV

C.felis_cf76-2 MMKFLLMFVVPLMTLAVDPEPVAAAQPQPAVTG---VQQVMPTNAPVVVTGQPASTPAVV

T.parva_p67 MQITQFLLIIPVFFVSAGDKMPTEEQPFPSRLSPLVTLESAITQPTPVYTMRSVGNVAKA

T.annulata_SPAG-1 MNIIHFLLTIPAIFVSGADKMPAGESSRTSKPSPLVTLESAVTQPS-KDPFKTISALSKA

B.bovis_BOV57 ------------------------------------MAFAKLSILFTFLLVHLVSTNAFD

C.felis_cf76-1 NQQSIPQAPNTPVVATGQDATDVRSITNVTAPTVQQPLPVQPPQQILQVPIVQQAVPAVQ

C.felis_cf76-2 NQQSIPQAPNTPVVATGQDATDVRSITNVTAPTVQQPLPVQPPQQILQVPIVQQAVPAVQ

T.parva_p67 AKVWKSAVSSPDVSTTIPTPVSEENITSTIHT-QTKEVPAASGSDSSTVTNLVQTQSQVQ

T.annulata_SPAG-1 TKVWKSAVSVSGDSKTVPTPVSEPMITRSFQEPVSQELEFQSDTEINESGSGSDEDEDDD

B.bovis_BOV57 LGEWSHDAHDTHD-------------IKAHHESAADATPGAAAQTLSESEEMEKALKALE

C.felis_cf76-1 PSAGIEVKDN---TSTAPANLENAITVPSVVPSVGSPSVPTTVP----------------

C.felis_cf76-2 PAGIEVKKDN---TSTAPANLENTTTVPSVVPAVGSPSVTTTVP----------------

T.parva_p67 DNVKQQQDTK------GNKTDSEEENEDSTVSTDVSPTIPTPVS----------------

T.annulata_SPAG-1 DDEEEEEDDKSTSSKNGKGSPKAQPGVSSSSTSSASPTSPTTTLSQTGLGPSGSHAQQDP

B.bovis_BOV57 EETKLENKPN---------------------------DTPTPVP----------------

C.felis_cf76-1 ------------------------------------------------------------

C.felis_cf76-2 ------------------------------------------------------------

T.parva_p67 ------------------------------------------------------------

T.annulata_SPAG-1 GVGVPGVGVPGVGVPGVGVPGVGVPGVGVPGVGGVPGVGVAPGVGVPGVGVAPGVGVGAD

B.bovis_BOV57 ------------------------------------------------------------

C.felis_cf76-1 ------------------------------------------------------------

C.felis_cf76-2 ------------------------------------------------------------

T.parva_p67 ------------------------------------------------------------

T.annulata_SPAG-1 SSGLPGSGGLGAGAKAGKGQGSGLQGPGGVGVVPGVGVAASSSSPGKPPGVGAGVMPGVG

B.bovis_BOV57 ------------------------------------------------------------

C.felis_cf76-1 -----------------------PTGVTTTQDRTNVPTAMEGSPPEVKTTVPVRVASEVQ

C.felis_cf76-2 -----------------------LPAVATTQDRTNVPTVVEASPPEVTS-----------

T.parva_p67 ----------------------EEIITPTLQAQTKEEVPPADLSDQVPSN----------

T.annulata_SPAG-1 VRAQGGVIIGAPGVAGVPGGKPGQPVSQELELKSDTEINESGSSSEGEDD----------

B.bovis_BOV57 -------------------------VTPSAEEKQDAPVE---------------------

C.felis_cf76-1 STSVPLAESSPPEVSPAVSSSNYLSQMGRATPGDRGGSIVTGPQVEP-----VADLREAE

C.felis_cf76-2 ------------SHSPAESSSNLLSQLGRATPGDRGGSIATGPQVETSSVKPAADLREAE

T.parva_p67 ------GSDSEEEDNKSTSSKDEKELKKTLQPGKTSTGETTSGQDLNSKQQQTGVSDLAS

T.annulata_SPAG-1 ------DDEEEEEENKSTSSKGAGGKAGKGQGSVSPGGGSSASQTSPTTTPQSGLASSGS

B.bovis_BOV57 ---------------ENKKVDQPKIEIPALHPPDSPLHTEKDDALDITTAPFTLVEDPAS

C.felis_cf76-1 GQVNRGGATPSG-MDGRNVTSGGILDGN---------IITDGLPIGINISASSESDIATS

C.felis_cf76-2 GQLNREGTTPSGRADGRNVTLGGMLDGN---------IITDGLPIGTNVSASSEGDITTS

T.parva_p67 GSHSSGLTVPGVGVPGAVSPQGGQSLASNTSREGQTQHQQARDGDGRVIEPKIGLPGSTS

T.annulata_SPAG-1 HAQQSPQQDPAPSKPSGGGVPGVGVPGVGVPGVG---VPGVGVAPGVGVVPGVGGATTSS

B.bovis_BOV57 HENELTSEIPQSPADDTNVNAGNEDSIIT---------DTTPIAKSMRLNTVTKIDETIE

C.felis_cf76-1 RILLGIGNEMSLIVDEILVKLEELKVLEDKKLVGNTQKLESLRESIITEYQK--------

C.felis_cf76-2 RILLGIGNEMSLIVDEILVKLEELKVLEDKKLVGNTQKLESLRESIITEYQK--------

T.parva_p67 APVPTRPPGSSTDTRPASSGPSAPGGPGSSSRSGGTRSTDSVTRPVPSPGAPGIIIRELG

T.annulata_SPAG-1 SSTTSTSTSTTTTTTTSSGKPSDQGSHGTSPRNAVTRQTDSISGPIPSPGDPRAITGQMG

B.bovis_BOV57 KLNHRLQTFLESVASS-AHDLTYYQSLLDTAYDIFCREINGDMSPMGSGGQLD-------

C.felis_cf76-1 -----FIQEITEIENSDENTKMDGIQSSDIAQTLRYKYDASVKNIMANVMKILNTKGKYD

C.felis_cf76-2 -----FIQEITEIENSDENTKMDGIQSSDIAQTLRYKYDASVKNIMANVMKILNTKGKYD

T.parva_p67 NRAMDIVQFLGRFKPEPRTYEGDRTNVAELKKFLFEELESLVNTLIELKLAIANDFVEIT

T.annulata_SPAG-1 EGERFAVQFLGDFKPKPRRYEGQGTDAVKLKQFIFEEVKSLVQTLINLKLAIANDFVEIS

B.bovis_BOV57 ----KDGNGVTLMISAEMSSAIRRSFDTKVEVLELAASEVASQKSKEVGAQTIHDALTVG

C.felis_cf76-1 GAILAYNYIKDKVQSIKNGIKNPSSEYLKLIRDIDFSADNIIDPMINNEEKVGIQLKDAK

C.felis_cf76-2 GAILAYNYIKDKVQSIKNGIKNPSSEYLKLIRDIDFSADNIIDPMINNEEKVGIQLKDAK

T.parva_p67 DGLRKNTKDHEARLKLLKGVEFTKRKSVANVVKGFSSLYCVLLMNMNGIKEKKRESEVAD

T.annulata_SPAG-1 EKLKKKNQNYVPKLKLLKGEQFDTKQKVANVLKGFNSLYFVFFMNLNLAKEVNKPEELAE

B.bovis_BOV57 LRTVRDTITSPGMTIHTTSNDMKNMTAIVADMSKGLLADIIKWTLKEDVLKKRLFDKIVE

C.felis_cf76-1 SKIFG-LLSNNTNNNITYDLKKKIIEHFNSLQEEHSIANSLINGAKKFSNKLEHLTNKLK

C.felis_cf76-2 SKIFG-LLSNNTNNNITYDLKKKIIEHFNSLQEEHSIANSLINGAKKFSNKLEHLTNKLK

T.parva_p67 GIWKLSTLPDKVANELLLAMEKIVVPPKTPELEEAFKAIEFGFKIAYYATKDILSSIENT

T.annulata_SPAG-1 FLWKLNTIPDKVGREFELAIEKTKGSEKKKELEEAFNSIGLGFKIAQYATNDILSSITNS

B.bovis_BOV57 RDNFIKTSPDELRMEAFTHAIRELAGEFHNAQKEKTGAIEKQNGFKEYMDEMREDINTIQ

C.felis_cf76-1 ISISKYVATADESNTIKFIHQASNALEKTNNTQIIMNTTNDSNAVKSTSDVQSMSVPLAE

C.felis_cf76-2 ISISKYVATADESNTIKFIHQASNALEKTNNTQIIMNTTNDSNAVKSTSDVQSMSVPLAE

T.parva_p67 VHNLMHAKNYEENFIAQVRNSLRMVPHQMNLTESSFVIKISDMMRRRGTASQDQPAGAGS

T.annulata_SPAG-1 VYSLIKLKNFGDDFVTEVRKSLQMVPHQKNLNGSAFIVKISEIINKKGTEDQDQTSGSG-

B.bovis_BOV57 RLIDTYFATVHKGHAKAILYEASKELRKDGNAESQLRLRVAEQKVHQEELKKAEP-----

C.felis_cf76-1 SSS----------NLLSQMGRATPRDRGGNEGSDGMKSSTGPQVEPAADLREAEGEVNKE

C.felis_cf76-2 SSS----------NLLSQMGRATPRDRGGNEGSDGMKSSTGPQVEPAADLREAEGEVNKE

T.parva_p67 AVTPGRGSSGTGRAAGTGGGSLRGLDLSEEEVKKILDEIVKDPSDGELGLGDLSDPSGRS

T.annulata_SPAG-1 -------------SKGTEGGSLRGQDLTEEEVLKVLDELVKDVSEEHVGIGDLSDPSSRT

B.bovis_BOV57 ---------------------------------KQEDTGCPYPTEP--------------

C.felis_cf76-1 ADGRNVTSGGEADGRNVTSGGKTSSLEDNTWNYGG-----INTENTKAKGNLKGKEEG--

C.felis_cf76-2 ADGRNVTSGGEADGRNVTSGGKTSSLEDNTWNYGG-----INTENTKAKGNLKGKEEG--

T.parva_p67 S-GRQPSLGPSLGITDGEAGPTIVSPTGPTIAAGG--EQPPSAPNGTAKGPAGTQPEG--

T.annulata_SPAG-1 PNAKPAELGPSLVIQNVPSDPSKVTPTQPSNLPQVPTTGPGNGTDGTTTGPGGNGEGGKD

B.bovis_BOV57 ------------------------------------------------------------

C.felis_cf76-1 -----ELKLVDDEDEEEAVKDGFNHIKIIATLLLSLTLV--

C.felis_cf76-2 -----ELKLVDDEDEEEAVKDGFNHIKIIATLLLSLTLV--

T.parva_p67 ---GEKKEGLIQKLKKKFLGSGFEVASLMIPMATIIISIVH

T.annulata_SPAG-1 LKEGEKKEGLFQKIKNKLLGSGFEVASIIIPMTTIIFSIVH

B.bovis_BOV57 -----------------------------------------
