# Supplementary material for: A Novel Candidate Vaccine for Cytauxzoonosis Inferred from Comparative Apicomplexan Genomics
Source: PLoS One. 2013 Aug 20;8(8):e71233. doi: 10.1371/journal.pone.0071233 (PMC3748084; doi:10.1371/journal.pone.0071233)
Supplement: Figure S4 — Amino acid sequences of syntenic gene C. felis cf76 from geographic isolates across the southeastern and southwestern United States (shading: black- identical amino acids, grey- similar amino acids). (DOC) [file pone.0071233.s004.doc]

► **-----------------------------------cf76 amino terminus fragment--------------------------------------**

AR1 1 MMKFLLMFVVPLMTLAVDPEPVAAAQPQPAVTGVQQVMPTNAPVVVTGQPASTPAVVNQQSIPQAPNTPV
MO 1 MMKFLLMFVVPLMTLAVDPEPVAAAQPQPAVTGVQQVMPTNAPVVVTGQPASTPAVVNQQSIPQAPNTPV
AR2 1 MMKFLLMFVVPLMTLAVDPEPVAAAQPQPAVTGVQQVMPTNAPVVVTGQPASTPAVVNQQSIPQAPNTPV
OK 1 MMKFLLMFVVPLMTLAVDPEPVAAAQPQPAVTGVQQVMPTNAPVVVTGQPASTPAVVNQQSIPQAPNTPV
OH 1 MMKFLLMFVVPLMTLAVDPEPVAAAQPQPAVTGVQQVMPTNAPVVVTGQPASTPAVVNQQSIPQAPNTPV
TN 1 MMKFLLMFVVPLMTLAVDPEPVAAAQPQPAVTGVQQVMPTNAPVVVTGQPASTPAVVNQQSIPQAPNTPV
KS 1 MMKFLLMFVVPLMTLAVDPEPVAAAQPQPAVTGVQQVMPTNAPVVVTGQPASTPAVVNQQSIPQAPNTPV
AR3 1 MMKFLLMFVVPLMTLAVDPEPVAAAQPQPAVTGVQQVMPTNAPVVVTGQPASTPAVVNQQSIPQAPNTPV
NC1 1 MMKFLLMFVVPLMTLAVDPEPVAAAQPQPAVTGVQQVMPTNAPVVVTGQPASTPAVVNQQSIPQAPNTPV
VA 1 MMKFLLMFVVPLMTLAVDPEPVAAAQPQPAVTGVQQVMPTNAPVVVTGQPASTPAVVNQQSIPQAPNTPV
NC2 1 MMKFLLMFVVPLMTLAVDPEPVAAAQPQPAVTGVQQVMPTNAPVVVTGQPASTPAVVNQQSIPQAPNTPV
consensus 1 **********************************************************************

 ----------------------------------------------------------------------------------------------------------------------------------------
AR1 71 VATGQDATDVRSITNVTAPTVQQPLPVQPPQQILQVPIVQQAVPAVQPAGIEVKKDNTSTAPANLENTTT
MO 71 VATGQDATDVRSITNVTAPTVQQPLPVQPPQQILQVPIVQQAVPAVQPAGIEVKKDNTSTAPANLENTTT
AR2 71 VATGQDATDVRSITNVTAPTVQQPLPVQPPQQILQVPIVQQAVPAVQPAGIEVKKDNTSTAPANLENTTT
OK 71 VATGQDATDVRSITNVTAPTVQQPLPVQPPQQILQVPIVQQAVPAVQPAGIEVKKDNTSTAPANLENTTT
OH 71 VATGQDATDVRSITNVTAPTVQQPLPVQPPQQILQVPIVQQAVPAVQPAGIEVKKDNTSTAPANLENTTT
TN 71 VATGQDATDVRSITNVTAPTVQQPLPVQPPQQILQVPIVQQAVPAVQPAGIEVKKDNTSTAPANLENTTT
KS 71 VATGQDATDVRSITNVTAPTVQQPLPVQPPQQILQVPIVQQAVPAVQPAGIEVKKDNTSTAPANLENTTT
AR3 71 VATGQDATDVRSITNVTAPTVQQPLPVQPPQQILQVPIVQQAVPAVQPAGIEVKKDNTSTAPANLENTTT
NC1 71 VATGQDATDVRSITNVTAPTVQQPLPVQPPQQILQVPIVQQAVPAVQPAGIEVKKDNTSTAPANLENTTT
VA 71 VATGQDATDVRSITNVTAPTVQQPLPVQPPQQILQVPIVQQAVPAVQPSAGIEVKDNTSTAPANLENAIT
NC2 71 VATGQDATDVRSITNVTAPTVQQPLPVQPPQQILQVPIVQQAVPAVQPSAGIEVKDNTSTAPANLENAIT
consensus 71 ************************************************ . ************* *

 ----------------------------------------------------------------------------------------------------------------------------------------
AR1 141 VPSVVPAVGSPSVTTTVPLPAVATTQDRTNVPTVVEASPPEVTS-----------------------SHS
MO 141 VPSVVPAVGSPSVTTTVPLPAVATTQDRTNVPTVVEASPPEVTS-----------------------SHS
AR2 141 VPSVVPAVGSPSVTTTVPLPAVATTQDRTNVPTVVEASPPEVTS-----------------------SHS
OK 141 VPSVVPAVGSPSVTTTVPLPAVATTQDRTNVPTVVEASPPEVTS-----------------------SHS
OH 141 VPSVVPAVGSPSVTTTVPLPAVATTQDRTNVPTVVEASPPEVTS-----------------------SHS
TN 141 VPSVVPAVGSPSVTTTVPLPAVATTQDRTNVPTVVEASPPEVTS-----------------------SHS
KS 141 VPSVVPAVGSPSVTTTVPLPAVATTQDRTNVPTVVEASPPEVTS-----------------------SHS
AR3 141 VPSVVPAVGSPSVTTTVPLPAVATTQDRTNVPTVVEASPPEVTS-----------------------SHS
NC1 141 VPSVVPAVGSPSVTTTVPLPAVATTQDRTNVPTVVEASPPEVTS-----------------------SHS
VA 141 VPSVVPSVGSPSVPTTVPPTGVTTTQDRTNVPTAMEGSPPEVKTTVPVRVASEVQSTSVPLAESSPPEVS
NC2 141 VPSVVPSVGSPSVPTTVPPTGVTTTQDRTNVPTAMEGSPPEVKTTVPVRVASEVQSTSVPLAESSPPEVS
consensus 141 ****** ****** **** .* ********** .*.***** . *

 ------------------------------------------------------**■** ►-------------------**cf76 central fragment-------------------**
AR1 188 PAESSSNLLSQLGRATPGDRG--GSIATGPQVETSSVKPAADLREAEGQLNREGTTPSGRADGRNVTLGGML
MO 188 PAESSSNLLSQLGRATPGDRG--GSIATGPQVETSSVKPAADLREAEGQLNREGTTPSGRADGRNVTLGGML
AR2 188 PAESSSNLLSQLGRATPGDRG--GSIATGPQVETSSVKPAADLREAEGQLNREGTTPSGRADGRNVTLGGML
OK 188 PAESSSNLLSQLGRATPGDRG--GSIATGPQVETSSVKPAADLREAEGQLNREGTTPSGRADGRNVTLGGML
OH 188 PAESSSNLLSQLGRATPGDRG--GSIATGPQVETSSVKPAADLREAEGQLNREGTTPSGRADGRNVTLGGML
TN 188 PAESSSNLLSQLGRATPGDRG--GSIATGPQVETSSVKPAADLREAEGQLNREGTTPSGRADGRNVTLGGML
KS 188 PAESSSNLLSQLGRATPGDRG--GSIATGPQVETSSVKPAADLREAEGQLNREGTTPSGRADGRNVTLGGML
AR3 188 PAESSSNLLSQLGRATPGDRG--GSIATGPQVETSSVKPAADLREAEGQLNREGTTPSGRADGRNVTLGGML
NC1 188 PAESSSNYLSLKSNATPVDRGLSEGNRIGPQ-----VEPVADLREAEGQLNREGTTPSGRADGRNVTLGGML
VA 211 PAVSSSNYLSQMGRATPGDRG—-GSIVTGPQ-----VEPVADLREAEGQVNRGGATPSG-MDGRNVTSGGIL
NC2 211 PAVSSSNYLSQMGRATPGDRG--GSIVTGPQ-----VEPVADLREAEGQVNRGGATPSG-MDGRNVTSGGIL
consensus 211 ** **** ** *** *** * * *********.** * **** ****** **.*

----------------------------------------------------------------------------------------------------------------------------------------
AR1 258 DGNIITDGLPIGTNVSASSEGDITTSRILLGIGNEMSLIVDEILVKLEELKVLEDKKLVGNTQKLESLRE
MO 258 DGNIITDGLPIGTNVSASSEGDITTSRILLGIGNEMSLIVDEILVKLEELKVLEDKKLVGNTQKLESLRE
AR2 258 DGNIITDGLPIGTNVSASSEGDITTSRILLGIGNEMSLIVDEILVKLEELKVLEDKKLVGNTQKLESLRE
OK 258 DGNIITDGLPIGTNVSASSEGDITTSRILLGIGNEMSLIVDEILVKLEELKVLEDKKLVGNTQKLESLRE
OH 258 DGNIITDGLPIGTNVSASSEGDITTSRILLGIGNEMSLIVDEILVKLEELKVLEDKKLVGNTQKLESLRE
TN 258 DGNIITDGLPIGTNVSASSEGDITTSRILLGIGNEMSLIVDEILVKLEELKVLEDKKLVGNTQKLESLRE
KS 258 DGNIITDGLPIGTNVSASSEGDITTSRILLGIGNEMSLIVDEILVKLEELKVLEDKKLVGNTQKLESLRE
AR3 258 DGNIITDGLPIGTNVSASSEGDITTSRILLGIGNEMSLIVDEILVKLEELKVLEDKKLVGNTQKLESLRE
NC1 255 DGNIITDGLPIGTNVSASSEGDITTSRILLGIGNEMSLIVDEILVKLEELKVLEDKKLVGNTQKLESLRE
VA 275 DGNIITDGLPIGINISASSESDIATSRILLGIGNEMSLIVDEILVKLEELKVLEDKKLVGNTQKLESLRE
NC2 275 DGNIITDGLPIGINISASSESDIATSRILLGIGNEMSLIVDEILVKLEELKVLEDKKLVGNTQKLESLRE
consensus 281 ************ *.***** ** **********************************************

 ----------------------------------------------------------------------------------------------------------------------------------------

AR1 328 SIITEYQKFIQEITEIENSDENTKMDGIQSSDIAQTLRYKYDASVKNIMANVMKILNTKGKYDGAILAYN
MO 328 SIITEYQKFIQEITEIENSDENTKMDGIQSSDIAQTLRYKYDASVKNIMANVMKILNTKGKYDGAILAYN
AR2 328 SIITEYQKFIQEITEIENSDENTKMDGIQSSDIAQTLRYKYDASVKNIMANVMKILNTKGKYDGAILAYN
OK 328 SIITEYQKFIQEITEIENSDENTKMDGIQSSDIAQTLRYKYDASVKNIMANVMKILNTKGKYDGAILAYN
OH 328 SIITEYQKFIQEITEIENSDENTKTDGIQSSDIAQTLRYKYDASVKNIMANVMKILNTKGKYDGAILAYN
TN 328 SIITEYQKFIQEITEIENSDENTKTDGIQSSDIAQTLRYKYDASVKNIMANVMKILNTKGKYDGAILAYN
KS 328 SIITEYQKFIQEITEIENSDENTKTDGIQSSDIAQTLRYKYDASVKNIMANVMKILNTKGKYDGAILAYN
AR3 328 SIITEYQKFIQEITEIENSDENTKTDGIQSSDIAQTLRYKYDASVKNIMANVMKILNTKGKYDGAILAYN
NC1 325 SIITEYQKFIQEITEIENSDENTKMDGIQSSDIAQTLRYKYDASVKNIMANVMKILNTKGKYDGAILAYN
VA 345 SIITEYQKFIQEITEIENSDENTKMDGIQSSDIAQTLRYKYDASVKNIMANVMKILNTKGKYDGAILAYN
NC2 345 SIITEYQKFIQEITEIENSDENTKMDGIQSSDIAQTLRYKYDASVKNIMANVMKILNTKGKYDGAILAYN
consensus 351 ************************ *********************************************

----------------------------------------------------------------------------------------------------------------------------------------

AR1 398 YIKDKVQSIKNGIKNPSSEYLKLIRDIDFSADNIIDPMINNEEKVGIQLKDAKSKIFGLLSNNTNNNITY
MO 398 YIKDKVQSIKNGIKNPSSEYLKLIRDIDFSADNIIDPMINNEEKVGIQLKDAKSKIFGLLSNNTNNNITY
AR2 398 YIKDKVQSIKNGIKNPSSEYLKLIRDIDFSADNIIDPMINNEEKVGIQLKDAKSKIFGLLSNNTNNNITY
OK 398 YIKDKVQSIKNGIKNPSSEYLKLIRDIDFSADNIIDPMINNEEKVGIQLKDAKSKIFGLLSNNTNNNITY
OH 398 YIKDKVQSIKNGIKNPSSEYLKLIRDIDFSADNIIDPMINNEEKVGIQLKDAKSKIFGLLSNNTNNNITY
TN 398 YIKDKVQSIKNGIKNPSSEYLKLIRDIDFSADNIIDPMINNEEKVGIQLKDAKSKIFGLLSNNTNNNITY
KS 398 YIKDKVQSIKNGIKNPSSEYLKLIRDIDFSADNIIDPMINNEEKVGIQLKDAKSKIFGLLSNNTNNNITY
AR3 398 YIKDKVQSIKNGIKNPSSEYLKLIRDIDFSADNIIDPMINNEEKVGIQLKDAKSKIFGLLSNNTNNNITY
NC1 395 YIKDKVQSIKNGIKNPSSEYLKLIRDIDFSADNIIDPMINNEEKVGIQLKDAKSKIFGLLSNNTNNNITY
VA 415 YIKDKVQSIKNGIKNPSSEYLKLIRDIDFSADNIIDPMINNEEKVGIQLKDAKSKIFGLLSNNTNNNITY
NC2 415 YIKDKVQSIKNGIKNPSSEYLKLIRDIDFSADNIIDPMINNEEKVGIQLKDAKSKIFGLLSNNTNNNITY
consensus 421 **********************************************************************

 -----------------------------------**■** ►**-------------------cf76 carboxy terminus region----------------------**
AR1 468 DLKKKIIEHFNSLQEEHSIANSLINGAKKFSNKLEHLTNKLKISISKYVATADESNTIKFIHQASNALEK
MO 468 DLKKKIIEHFNSLQEEHSIANSLINGAKKFSNKLEHLTNKLKISISKYVATADESNTIKFIHQASNALEK
AR2 468 DLKKKIIEHFNSLQEEHSIANSLINGAKKFSNKLEHLTNKLKISISKYVATADESNTIKFIHQASNALEK
OK 468 DLKKKIIEHFNSLQEEHSIANSLINGAKKFSNKLEHLTNKLKISISKYVATADESNTIKFIHQASNALEK
OH 468 DLKKKIIEHFNSLQEEHSIANSLINGAKKFSNKLEHLTNKLKISISKYVATADESNTIKFIHQASNALEK
TN 468 DLKKKIIEHFNSLQEEHSIANSLINGAKKFSNKLEHLTNKLKISISKYVATADESNTIKFIHQASNALEK
KS 468 DLKKKIIEHFNSLQEEHSIANSLINGAKKFSNKLEHLTNKLKISISKYVATADESNTIKFIHQASNALEK
AR3 468 DLKKKIIEHFNSLQEEHSIANSLINGAKKFSNKLEHLTNKLKISISKYVATADESNTIKFIHQASNALEK
NC1 465 DLKKKIIEHFNSLQEEHSIANSLINGAKKFSNKLEHLTNKLKISISKYVATADESNTIKFIHQASNALEK
VA 485 DLKKKIIEHFNSLQEEHSIANSLINGAKKFSNKLEHLTNKLKISISKYVATADESNTIKFIHQASNALEK
NC2 485 DLKKKIIEHFNSLQEEHSIANSLINGAKKFSNKLEHLTNKLKISISKYVATADESNTIKFIHQASNALEK
consensus 491 **********************************************************************

 ----------------------------------------------------------------------------------------------------------------------------------------
AR1 538 TNNTQIIMNTTNDSNAVKSTSDVQSMSVPLAESSSNLLSQMGRATPRDRGGNEGSDGMKSSTGPQVEPAA
MO 538 TNNTQIIMNTTNDSNAVKSTSDVQSMSVPLAESSSNLLSQMGRATPRDRGGNEGSDGMKSSTGPQVEPAA
AR2 538 TNNTQIIMNTTNDSNAVKSTSDVQSMSVPLAESSSNLLSQMGRATPRDRGGNEGSDGMKSSTGPQVEPAA
OK 538 TNNTQIIMNTTNDSNAVKSTSDVQSMSVPLAESSSNLLSQMGRATPRDRGGNEGSDGMKSSTGPQVEPAA
OH 538 TNNTQIIMNTTNDSNAVKSTSDVQSMSVPLAESSSNLLSQMGRATPRDRGGNEGSDGMKSSTGPQVEPAA
TN 538 TNNTQIIMNTTNDSNAVKSTSDVQSMSVPLAESSSNLLSQMGRATPRDRGGNEGSDGMKSSTGPQVEPAA
KS 538 TNNTQIIMNTTNDSNAVKSTSDVQSMSVPLAESSSNLLSQMGRATPRDRGGNEGSDGMKSSTGPQVEPAA
AR3 538 TNNTQIIMNTTNDSNAVKSTSDVQSMSVPLAESSSNLLSQMGRATPRDRGGNEGSDGMKSSTGPQVEPAA
NC1 535 TNNTQIIMNTTNDSNAVKSTSDVQSMSVPLAESSSNLLSQMGRATPRDRGGNEGSDGMKSSTGPQVEPAA
VA 555 TNNTQIIMNTTNDSNAVKSTSDVQSMSVPLAESSSNLLSQMGRATPRDRGGNEGSDGMKSSTGPQVEPAA
NC2 555 TNNTQIIMNTTNDSNAVKSTSDVQSMSVPLAESSSNLLSQMGRATPRDRGGNEGSDGMKSSTGPQVEPAA
consensus 561 **********************************************************************

--------------------- **REPEAT REPEAT -**------------------------------------------------------------------------
AR1 608 DLREAEGEVNK**EADGRNVTSG**G**EADGRNVTSG**GKTSSLEDNTWNYGGINTENTKAKGNLKGKEEGELKLV
MO 608 DLREAEGEVNK**EADGRNVTSG**-**----------**GKTSSLEDNTWNYGGINTENTKAKGNLKGKEEGELKLV
AR2 608 DLREAEGEVNK**EADGRNVTSG**G**EADGRNVTSG**GKTSSLEDNTWNYGGINTENTKAKGNLKGKEEGELKLV
OK 608 DLREAEGEVNK**EADGRNVTSG**G**EADGRNVTSG**GKTSSLEDNTWNYGGINTENTKAKGNLKGKEEGELKLV
OH 608 DLREAEGEVNK**EADGRNVTSG**G**EADGRNVTSG**GKTSSLEDNTWNYGGINTENTKAKGNLKGKEEGELKLV
TN 608 DLREAEGEVNK**EADGRNVTSG**G**EADGRNVTSG**GKTSSLEDNTWNYGGINTENTKAKGNLKGKEEGELKLV
KS 608 DLREAEGEVNK**EADGRNVTSG**G**EADGRNVTSG**GKTSSLEDNTWNYGGINTENTKAKGNLKGKEEGELKLV
AR3 608 DLREAEGEVNK**EADGRNVTSG**G**EADGRNVTSG**GKTSSLEDNTWNYGGINTENTKAKGNLKGKEEGELKLV
NC1 605 DLREAEGEVNK**EADGRNVTSG**G**EADGRNVTSG**GKTSSLEDNTWNYGGINTENTKAKGNLKGKEEGELKLV
VA 625 DLREAEGEVNK**EADGRNVTSG**G**EADGRNVTSG**GKTSSLEDNTWNYGGINTENTKAKGNLKGKEEGELKLV
NC2 625 DLREAEGEVNK**EADGRNVTSG**G**EADGRNVTSG**GKTSSLEDNTWNYGGINTENTKAKGNLKGKEEGELKLV
consensus 631 ************************* **************************************


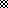

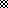


----------------------------------------------------- **■**
AR1 678 DDEDEEEAVKDGFNHIKIIATLLLSLTLV
MO 667 DDEDEEEAVKDGFNHIKIIATLLLSLTLV
AR2 678 DDEDEEEAVKDGFNHIKIIATLLLSLTLV
OK 678 DDEDEEEAVKDGFNHIKIIATLLLSLTLV
OH 678 DDEDEEEAVKDGFNHIKIIATLLLSLTLV
TN 678 DDEDEEEAVKDGFNHIKIIATLLLSLTLV
KS 678 DDEDEEEAVKDGFNHIKIIATLLLSLTLV
AR3 678 DDEDEEEAVKDGFNHIKIIATLLLSLTLV
NC1 675 DDEDEEEAVKDGFNHIKIIATLLLSLTLV
VA 695 DDEDEEEAVKDGFNHIKIIATLLLSLTLV
NC2 695 DDEDEEEAVKDGFNHIKIIATLLLSLTLV
consensus 701 *****************************
